# Novel Apo E-Derived ABCA1 Agonist Peptide (CS-6253) Promotes Reverse Cholesterol Transport and Induces Formation of preβ-1 HDL *In Vitro*

**DOI:** 10.1371/journal.pone.0131997

**Published:** 2015-07-24

**Authors:** Anouar Hafiane, John K. Bielicki, Jan O. Johansson, Jacques Genest

**Affiliations:** 1 Cardiovascular Research Laboratories Laboratory, Research Institute of the McGill University Health Centre, Montréal, Québec H4A 3J1, Canada; 2 Lawrence Berkeley National Laboratory, Donner Laboratory, MS1-267, Berkeley, CA, United States of America; 3 Artery Therapeutics, San Ramon, CA, United States of America; Wake Forest School of Medicine, UNITED STATES

## Abstract

Apolipoprotein (apo) mimetic peptides replicate some aspects of HDL function. We have previously reported the effects of compound ATI-5261 on its ability to replicate many functions of native apo A-I in the process of HDL biogenesis. ATI-5261 induced muscle toxicity in wild type C57Bl/6 mice, increased CPK, ALT and AST and increase in triglyceride (Tg) levels. Aromatic phenylalanine residues on the non-polar face of ATI-5261, together with positively charged arginine residues at the lipid-water interface were responsible for these effects. This information was used to create a novel analog (CS-6253) that was non-toxic. We evaluated this peptide designed from the carboxyl terminus of apo E, in its ability to mimic apo A-I functionality. Our data shows that the lipidated particles generated by incubating cells overexpressing ABCA1 with lipid free CS-6253 enhances the rate of ABCA1 lipid efflux with high affinity interactions with native ABCA1 oligomeric forms and plasma membrane micro-domains. Interaction between ABCA1 and lipid free CS-6253 resulted in formation of nascent HDL-CS-6253 particles that are actively remodeled in plasma. Mature HDL-CS-6253 particles deliver cholesterol to liver cells via SR-BI *in-vitro*. CS-6253 significantly increases cholesterol efflux in murine macrophages and in human THP-1 macrophage-derived foam cells expressing ABCA1. Addition of CS-6253 to plasma dose-dependently displaced apo A-I from α-HDL particles and led to *de novo* formation of preβ-1 HDL that stimulates ABCA1 dependent cholesterol efflux efficiently. When incubated with human plasma CS-6253 was also found to bind with HDL and LDL and promoted the transfer of cholesterol from HDL to LDL predominantly. Our data shows that CS-6253 mimics apo A-I in its ability to promote ABCA1-mediated formation of nascent HDL particles, and enhances formation of preβ-1 HDL with increase in the cycling of apo A-I between the preβ and α-HDL particles *in-vitro*. These mechanisms are potentially anti-atherogenic.

## Introduction

Reverse cholesterol transport (RCT) is considered to be the major beneficial effect of high density lipoproteins (HDL), through its major protein moiety apolipoprotein (apo) apo A-I [1]. Modulation of the RCT pathway may provide a therapeutic target for the prevention and treatment of atherosclerotic cardiovascular disease (CVD). Despite the recent failure of therapies designed to raise HDL-cholesterol in humans, a new approach to therapy using mimetics of HDL and its components continues to show promise. Although numerous apoA-I mimetics are under investigations [2], a single ‘best’ peptide that mimics all of the properties of apoA-I has not been identified [3, 4]. Moreover, the mechanisms by which these peptides exert an influence on HDL structure and metabolism are not completely understood [5]. Recently, novel engineered apolipoprotein mimetic peptides are being investigated as possible therapeutic molecules [6]. These peptides do not necessarily have sequence homology to apo A-I protein but mimic many of its physiological effects [7–9], thereby offering an approach to modulate HDL for therapeutic purposes We have previously reported the effects on apo E mimetic peptide ATI-5261 on its ability to replicate many of the functions of native apo A-I in the process of HDL biogenesis. However, in contrast to CS-6253, ATI-5261 induced muscle toxicity in wild-type C57Bl/6 and transiently increased CPK, ALT and AST activities, and plasma Tg levels. This information was used to create a novel analog. In the present study, we evaluated a mono-helical, 26-amino acid peptide (CS-6253) in its ability to mimic apo A-I functionality in the RCT process *in-vitro*. The peptide was developed in an iterative screening process utilizing ABCA1 mediated cholesterol cell screens and safety screens. Since CS-6253 represents a safe and drugable compound, we investigated its anti-atherogenic effects on different stages of RCT, including ABCA1 interactions, HDL assembly and subsequent HDL remodeling events in human plasma CS-6253 is a close analog of ATI-5261, with substitutions of aromatic phenylalanine for aliphatic leucine residues [7] and has a superior safety profile. It retains the structural features and high aqueous solubility of ATI-5261 [10], but its physiological effects have yet to be reported. Like ATI-5261, peptide CS-6253 was designed using determinants from the carboxyl terminal lipid binding domains of apo A-I and apo E necessary for mediating cellular lipid efflux and HDL formation [7, 11]. By using BHK cells, murine macrophages and human THP-1 cells expressing ABCA1 we investigated several CS-6253-mediated steps through our *in-vitro* model of HDL biogenesis and RCT using native human apo A-I as control. We addressed the question whether this peptide mediates RCT key steps via ABCA1 dynamics and found that functional HDL-CS-6253 lipoprotein particles were generated after peptide interaction with ABCA1 and plasma membrane (PM) microdomains. We further determined that CS-6253 mimics apo A-I in promoting HDL remodelling *in-vitro*. Importantly, we investigated the ability of CS-6253 to increase formation of pre-β HDL particles in plasma. Our data supports the concept that CS-6253 has potential therapeutic applications

### Experimental procedures

We carried out experiments on CS-6253 using similar protocols initially, as those reported for ATI-5261. For comparisons of ATI-5261 and CS-6253, vs. controls, experiments were run simultaneously under identical conditions so that specific kinetic parameters could be compared. Methods and results can be found on the on-line Supplementary appendix of this article, S1 Appendix. Where protocols differ, these are presented here.

### Apo E mimetic peptide

The CS-6253 peptide was synthesized by (Biosynthesis Inc., Lewisville TX) from all L-amino acids and capped with N-terminal acetyl and carboxyl-terminal amide groups. They form a class A amphipathic α-helix common to those found in apo A-I and apo E. CS-6253 is a modification of peptide ATI-5261 with a substitution of phenylalanine for leucine residues, and arginine for citrulline residues. For experiments, lyophilized peptide was dissolved in 10 mM phosphate buffer (pH 7.4) saline (PBS) (150 mM NaCl), filter sterilized (0.2 μm Nalgene capsules), and stored at 4°C until use.

### Animals

The mice toxicity study protocol was approved and performed according to Veterans Administration Palo Alto Health Care System Institutional Animal Care and Use Committee (IACUC), approval study number AZH1155. The mice toxicity studies were conducted in chow fed mice (male, C57BL/6) eight to ten-week old with intraperitoneal injection of 300 mg/kg CS-6253 and ATI-5261 peptides and PBS. Peptide CS-6253 was assessed for muscle toxicity with, ATI-5261 as positive and PBS as negative control respectively. Blood was collected using retro-orbital eye bleeding at 4 and 6 hours while the animals were under isoflurane.

### Toxicology

Creatine phosphokinase, aspartate- and alanine-aminotransferase activities (CPK, AST and ALT, respectively) in serum were quantified by IDEXX Laboratories, Inc. (Maine) to assess liver toxicity/necrosis. Tg levels in serum were determined using commercial kits

### Cell culture systems

BHK cells stably transfected with an ABCA1 or ABCG1 expression vector that is mifepristone inducible and cells transfected with the same vector lacking the ABCA1 cDNA insert (mutant or mock-transfected) were generously provided by the late Dr. John F. Oram from the Department of Medicine, University of Washington, and were characterized and cultured as previously described [12, 13]. These BHK-mock cells do not express ABCA1 and were used as controls. Mouse-derived peritoneal macrophages J774 were purchased from American Tissue Culture Collection (ATCC TIB-67, Cederlane, Burlington, Canada). For induction of ABCA1, BHK cells were incubated for 18–20 h with 10 nM mifepristone. J774 macrophages were stimulated with 0.3 mM Cpt-cAMP 8-(4-Chlorophenylthio)-cyclic adenosine monophosphate (Sigma). All BHK and J774 macrophages cells were respectively grown and maintained in DMEM and RPMI containing 10% fetal serum until experimental treatment. Studies were carried out on standard 24-well plates (150,000 cells/well) unless stated otherwise

### Foam cell formation

Human THP-1 macrophages (American Type Tissue Culture Collection, Camden, NJ) were grown as previously reported [14]. Cells were plated at 2x10^5^/ml 24 well-plates in RPMI1640 medium containing 10% fetal bovine serum (FBS), 50 μM β–mercaptoethanol, 50 μg/ml gentamicin, and maintained at 37°C in a humidified atmosphere of 5% CO_2_. THP-1 cells were differentiated into macrophages by the addition of 200 nM phorbol myristyl acetate (PMA) for 72h. Macrophages were incubated for 72h to become fully differentiated macrophages prior to experiment. Macrophages were transformed into foam cells by incubation with the presence of 100 μg/ml acetylated low-density lipoprotein (AcLDL) in serum-free RPMI 1640 medium containing 1% bovine serum albumin (BSA)

### Competitive cell binding assays

Competition binding assays were performed as described previously [15, 16]. Briefly, apo A-I was iodinated with ^125^I by Iodo-Gen (Pierce) to a specific activity of ~ 4000–3000 cpm/ng apo A-I. BHK cells were grown on 24-well plates and were stimulated with 10 nM mifepristone for 18–20h (Sigma-Aldrich, Oakville, Ontario, Canada). Cells were then incubated at 37°C with ^125^I-apo A-I (2μg/ml) in DMEM/BSA (1 mg/ml) in the presence of increasing amounts of either lipid free CS-6253, apo E or unlabeled lipid free apo A-I for 2 h. The cells were then washed rapidly twice with ice-cold PBS/BSA and twice with cold PBS and lysed with 0.1 N NaOH. The amount of bound iodinated ligand was determined by γ counting. The protein concentration in each well was determined using the Lowry assay, with albumin as the standard [17]. Control experiments were conducted to examine whether the apparent decrease in cell binding of the labeled apo A-I may be attributable to the ^125^I-apo A-I binding to different competitor particles instead of the cells. Therefore, an experiment was carried out in which CS-6253 particles were incubated with ^125^I-apo A-I under similar conditions used for the apo A-I binding assay and then the CS-6253 sample was separated by fast-protein liquid chromatography (FPLC). No significant amount of ^125^I-apo A-I was found associated with CS-6253 or aggregated in media (data not shown).

### Efflux assays

We investigated cholesterol efflux from BHK cells after adding lipid free CS-6253 to media cell culture or to human EDTA-plasma which had been stored at -80°C. BHK overexpressing human ABCA1 were labeled with 2 μCi/ml ^3^H-free cholesterol (Perkin Elmer, Norwalk, Connecticut) for 24 h. Afterward, BHK-ABCA1 were stimulated with 10 nmol/l mifepristone for 18–20 h (13). Cells were incubated with various doses of acceptor in media for 4 h at 37°C. Alternatively, plasma samples were first pre-incubated for 5 min at 37°C in plasma at mole ratios (10:1 or 1:1 relative to plasma apo A-I). To obtain the HDL fraction, apoB lipoproteins were precipitated from plasma by polyethylene glycol (PEG) [18]. Also, increased doses of lipid free CS-6253 and nHDL-CS-6253 were tested for cholesterol efflux after incubation with human plasma for optimum 1h at 37°C. ^3^[H]-cholesterol loaded BHK cells expressing ABCA1 were incubated with 2.8% apoB depleted plasma samples [19] for 4 h for the efflux assays. Lipid free apo A-I and nHDL-apo A-I were used as control, nHDL-CS-6253 and nHDL-apo A-I were generated after incubation with BHK cells expressing ABCA1. Direct comparisons with peptide ATI-5261 are provided using identical protocols (12).

Human macrophages THP-1 cells were labelled with ^3^H-cholesterol (5 μCi/ml) incorporated into AcLDL (100 μg/ml) in the presence of 1% FBS, 50 μM β-mercaptoethanol and 200 nM PMA, and incubated for 72h. Monolayers were extensively washed with serum free RPMI before exposure to CS-6253 or apoA-I at 0.96 μM for 24h. Medium and cell associated ^3^H-cholesterol were then measured by liquid scintillation counting. Cholesterol efflux was calculated according to the following equation as the percentage of ^3^H-cpm medium / (^3^H-cpm medium + ^3^H-cpm cells) × 100%. Alternatively, prior to efflux, THP-1 cell lipids were extracted with hexane: isopropanol (3v/2v) (1h, room temperature), the samples were loaded onto Silica Gel (Newark, US) thin layer chromatography plate (TLC). Bands corresponding to ^3^[H]-FC and ^3^[H]-CE were located by exposure to iodine vapor, and were scraped off the plate into liquid scintillating vials and assayed for radioactivity.

### Lipid labeling

Lipid labeling was performed as described previously [20, 21] with minor modifications. Briefly, BHK-cells were labeled with either 5 μCi/ml ^3^[H] choline (Perkin-Elmer) for 48 h or 3 μCi/ml ^3^[H] cholesterol (Perkin-Elmer) for 24 h, and then stimulated as described above. Cells were subsequently incubated with lipid-free apoA-I or CS-6253 at (0.96 μM) for 12h, and fractionated by sucrose gradient in the presence of 0.2% Triton X-100. For each fraction, lipids were extracted by Folch and ^3^[H]choline-labeled PtdCho, and SM were separated by TLC in the solvent system chloroform-methanol-water (65:35:4; v/v/v), and counted.

### Lipoprotein Preparation

Blood samples were obtained from healthy control subjects after a 12 h fast. Isolated plasma was kept in ice until separation of lipoproteins or was frozen at −80°C until analysis of lipids and apolipoproteins. All participants gave verbal consent for the donation of blood (approximately 15 mL) for research purposes. Participants were volunteers from the research laboratory. A log book containing the volunteer’s donor ID and health care card number is kept by a research nurse. The nurse collecting the blood and participant both sign the record and participant’s documents are kept in a registry. The consent procedure of our protocol for research on human subjects was reviewed and accepted by the Research Ethics Board of the McGill University Health Center (REB 10–208). Plasma apoA-I concentrations were measured by nephelometry (Behring Nephelometer 100 Analyzer). Human VLDL (d = 1.006 g/ml), (LDL (1.019<d<1.063 g/mL) and HDL (1.09<d<1.21 g/mL) were isolated by sequential ultracentrifugation, [22] using KBr as a salt in a 50.4 Ti rotor (Beckman Instruments, Palo Alto, CA). Isolated VLDL, LDL and HDL fractions were then extensively dialyzed against PBS, and total protein concentrations in these fractions were measured using a modified Bradford procedure (BioRad, Mississauga, Canada). Human AcLDL were purchased from Alfa Aesar (Ward Hill, USA)

### Lipoprotein separation by two-dimensional gel electrophoresis

**Fig 1:**
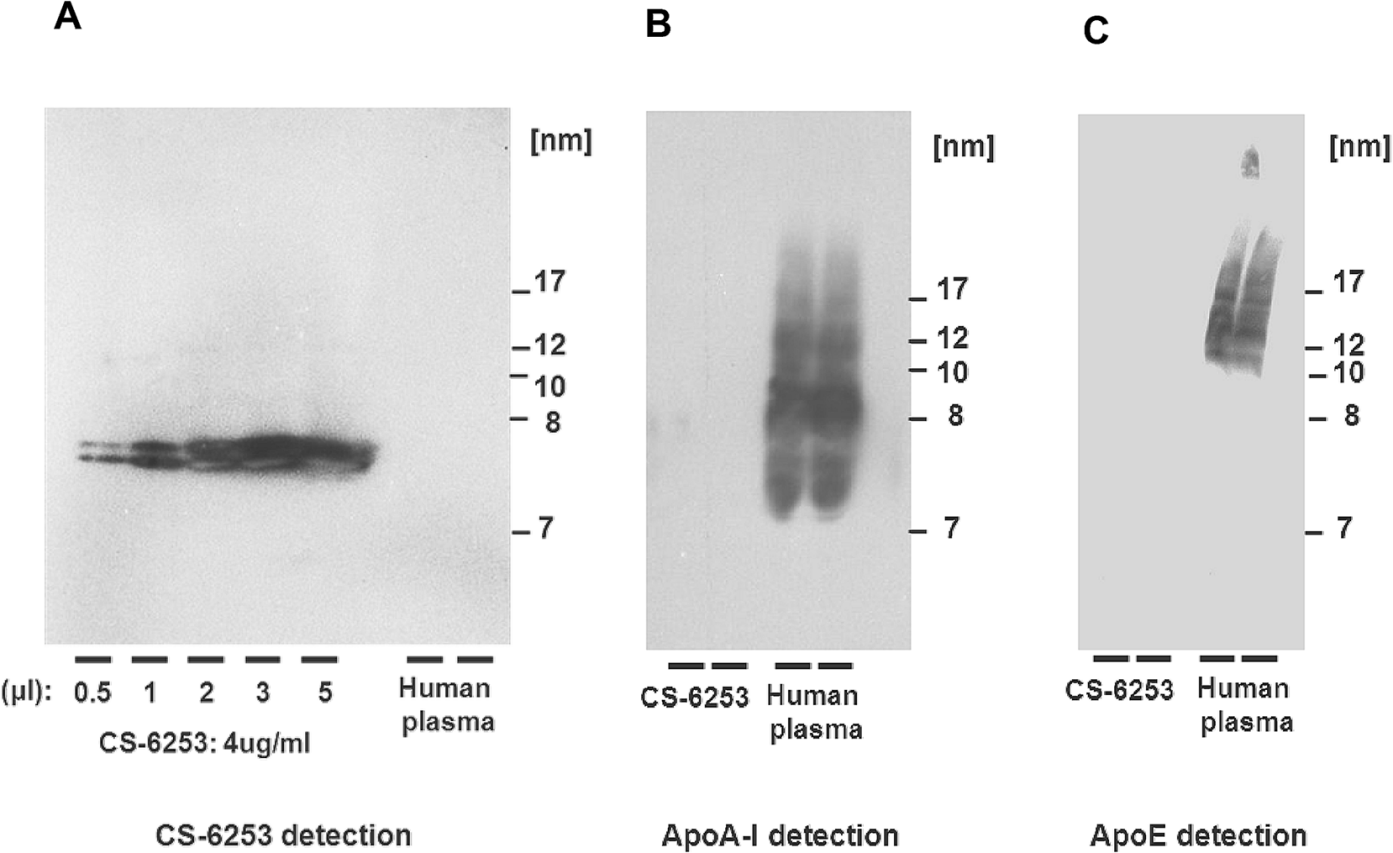
Cross reactivity characterisation of anti-CS-6253 antibody. Non denaturing gradient gel electrophoresis (4–20%) is used to separate CS-6253 complexes, apo A-I and apo E. CS-6253 was immunoblotted using antibody against human apo A-I, human apo E and against CS-6253. Normolipidemic human plasma was used as positive control. Molecular weight markers were revealed by Ponceau S. Left panel shows dose response relationship with an antibody directed against CS-6253. Middle panel shows anti-apo A-I antibody does not cross-react with CS-6253; similarly, right panel shows antibody against apo E does not react against CS-6253.

Apo A-I and CS-6253-containing particles were separated according to charge (agarose, horizontal axis) and size (polyacrylamide gradient, 3% to 20% gel, vertical axis) as previously described [23]. Apo A-I-containing particles were detected by incubating the membranes with immunopurified polyclonal anti-apo A-I antibody (Biodesign). The nHDL-CS-6253 containing particles were detected with immunopurified polyclonal anti-CS-6253 antibody (Biosynthesis Inc., Lewisville, TX). The anti-CS-6253 is a purified rabbit IgG polyclonal antibody specifically produced by Biosynthesis Inc. It is. Cross reactivity of this antibody was verified by Nondenaturing gradient gel electrophoresis (ND-PAGGE) 5% to 35% [24]. The anti-CS-6253 antibody recognizes only CS-6253 peptide in plasma; neither apo A-I nor apo E were detected by this antibody after separation by gradient gel electrophoresis respectively (**Fig 1A, 1B and 1C**). High molecular weight protein mixture (GE Healthcare, UK) was run as a standard on each gel. Molecular weight markers were revealed by Ponceau S sodium salt.

### FPLC analysis

Lipoprotein profiles were obtained using FPLC separation with a Superose 6B column (GE Healthcare, Madison, WI). A 200 μl aliquot of concentrated medium by ultrafiltration (spiral ultrafiltration cartridge, MWCO 50,000, Amicon), or 50 μl of plasma samples treated with CS-6253 were loaded onto the column, and eluted with TS buffer (50 mM Tris, 0.15 M NaCl, pH 7.4) at a constant flow rate of 0.35 ml/min. An aliquot of 250 μl from each fraction (0.5 ml) was used for the determination of radioactivity. Fractions corresponding to elution peaks were analyzed by Western blotting to detect apo A-I or CS-6253. The total cholesterol in plasma was determined enzymatically by the use of a commercial kit (ThermoElectron, Waltham, MA)

### Human plasma apo A-I and apo E

Purified human plasma apo A-I (Biodesign) and apo E (Biodesign) were resolubilized in 4 M guanidine-HCL and dialyzed extensively against Tris buffer, (10 mm Tris, 150 mm NaCl; pH 8.2) [16, 25]. Freshly solubilized apo A-I and apo E were used within 48 h

### SR-BI receptor-mediated cholesterol uptake

J774 cells were labeled for 24h with ^3^[H]-cholesterol (Perkin Elmer, Norwalk, Connecticut) in medium containing 1% FBS. Unlabeled rat Fu5AH hepatoma cells that express high levels of SR-BI, were prepared as described previously [26]. Fu5AH were first seeded on 24-well plate at a density of 2x10^5^ cells/well in DMEM plus 10% FBS. SR-BI mediated cholesterol uptake is determined as previously reported [27]. The nHDL particles were generated in media cell culture after 6h incubation of cAMP stimulated J774 cells with increased molar concentrations relative to native apo A-I of lipid free CS-6253 (0, 0.018, 0.071, 0.179, 0.357, 0.536, 0.714, and 1.071 μM). To prevent cholesterol esterification, 2 μg/ml of the ACAT inhibitor (CP113,818) was added during labeling, equilibration, and the efflux stages of the experiment. To inhibit *de novo* cholesterol synthesis, the HMG-CoA reductase inhibitor Mevinolin was added to the efflux medium (5 μg/ml) [28]. To assess SR-BI-mediated lipid uptake, ^3^[H]cholesterol labeled HDL particles (nHDL-CS-6253 and nHDL-apo A-I) were first incubated in normolipidemic human plasma for 1 hour incubation at 37°C [20]. Plasma containing lipidated radiolabelled HDL-peptide and HDL-apo A-I were obtained after PEG precipitation of apoB particles [18]. Thereafter, samples volumes of 20% apoB depleted plasma were incubated for 6h with Fu5AH cells expressing SR-BI [29]. Fu5AH cells were then washed 3 times with cold PBS, and the cell lipids were extracted with isopropyl alcohol as previously described [30]. The total of ^3^H-label present in the lipid extract from cells (% cpm) was quantified by liquid scintillation, and plotted as cell-associated radioactivity as described by Yancey et al. [30]. The contribution of SR-BI to the influx of HDL cholesterol (HDL-C) was assessed by a 2h pretreatment of Fu5AH cells with blocker of lipid transport-1 (BLT-1) to inhibit any transport of cholesterol from HDL to the cells via SR-BI [31]. The integrity and quality of lipidation of HDL mimetic particles were verified by 2D-PAGGE analysis performed within 24 h

### Statistical analysis

Data are shown as mean ± standard deviation (SD). Results were compared statistically by 2-tailed Student’s *t*-test and p values <0.05 were considered significantly significant. Michaelis-Menten curve fitting was used to determine the kinetics of binding, cell association and lipid efflux. The Lineweaver–Burk plot method was also used to determine cholesterol efflux kinetics for treatment and correspondent control condition. The data was analyzed with the Graph-Pad Prism6 software (GraphPad Software, Inc. La Jolla, CA), and expressed as mean ± standard deviation (SD)

## Results

### Toxicity

Toxicity was assessed in C57Bl/6 mice 4h following 300mg/kg intra-peritoneal injection (300 mg/kg) of ATI-5261, CS-6253 and vehicle (PBS) showing induced muscle toxicity by ATI-5261 as illustrated by serum CPK (31848.75±5583.89, p = 0.001; 153.75±33.45, p = 0.31; and 116.25±56.83 IU/L, respectively), ALT (239.50±54.19, p = 0.004; 36.25±3.77, p = 0.75;and 37.75±7.89 IU/L, respectively), and AST (3640±959.36, p = 0.005; 68.25±3.50, p = 0.03; and 52.75±8.50 IU/L, respectively). Parallely plasma Tg levels were measured showing reduce in mice (n = 6) injected with CS-6253. Plasma Tg levels in ATI-5261, CS-6253, and vehicles were as follows: (3201.96±168.48, p = 0.000041; 321.27±78.65, p = 0.0065; and 57.41±7.65, respectively).

### CS-6253 mediates cellular cholesterol efflux from ABCA1 in a time-dependent manner

**Fig 2:**
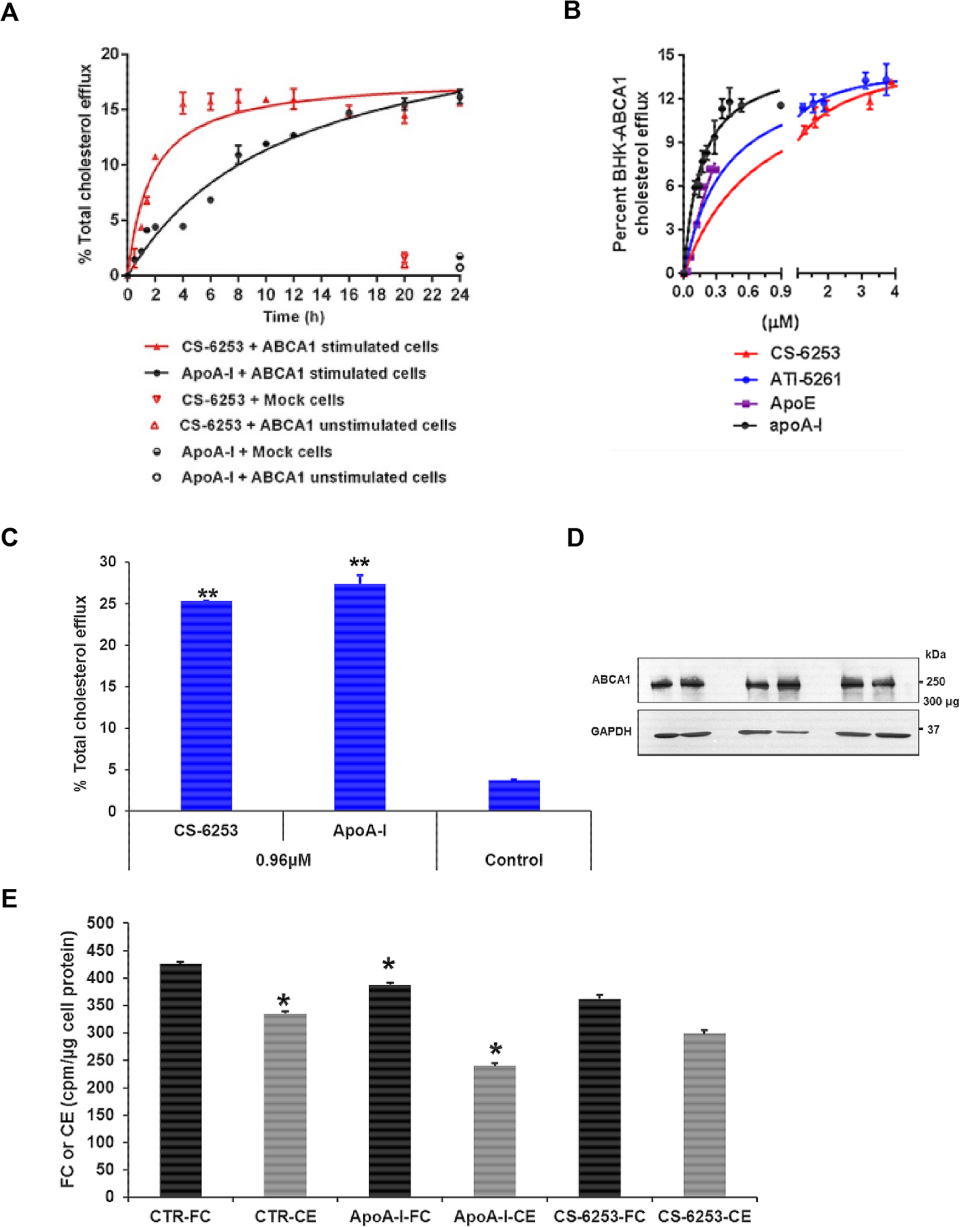
Effect of CS-6253 treatment on cholesterol efflux. A. Time-course activity of ABCA1-mediated cholesterol efflux to lipid-free CS-6253 (30 μg/ml) and apo A-I (10 μg/ml), using BHK cells expressing ABCA1, and control cells (unstimulated BHK-ABCA1 and BHK-mock cells). Cholesterol efflux from either ABCA1 cells or control cells to apo A-I (closed circles), and CS-6253 (closed triangles) was determined at the indicated time points. B. Dose dependent ABCA1 mediated cholesterol efflux in BHK-ABCA1 induced with mifepristone. Kinetic parameters for ABCA1-mediated cholesterol efflux from BHK-ABCA1 cells to apo A-I: *K_m_* = 4.53±0.67 μg/ml (0.15±0.02 μM), *V_max_* = 14.85±0.02% efflux/4h, and relative catalytic efficiency: *V_max_/K_m_* = 3.27. CS-6253: *K_m_* = 2.27±0.16 μg/ml (0.73±0.05 μM), *V_max_* = 15.25±0.05% efflux/4h, and relative catalytic efficiency: *V_max_/K_m_* = 6.71. ATI-5261: *K_m_* = 1.04 ± 0.16 μg/ml (0.37 ± 0.04 μM), *V_max_* = 14.48 ± 0.29% efflux/ h, and relative catalytic efficiency: *V_max_/K_m_* = 13.92. Apo E: *K_m_* = 10.30±2.84 μg/ml (0.21±0.05 μM), *V_max_* = 11.45±1.16% efflux/4h, and relative catalytic efficiency: *V_max_/K_m_* = 1.11. C. Ability of CS-6253 to stimulate cholesterol efflux from human macrophages THP-1. Foam cells were incubated with CS-6253 or apoA-I at equimolar ratio (0.96 μM) for 4h. Cholesterol efflux induced by CS-6253 and apoA-I were compared to control cells incubated alone. P<0.001 by Student’s t-test. D. Membrane ABCA1 levels assessment by Western blotting assays. ABCA1 was detected from THP-1 foam cells lysis at 4°C with lysis buffer containing 0.5% n-dodecylmaltoside in the presence of a protease inhibitor mixture followed by low speed centrifugation to remove cell debris. Protein concentration was determined by standard assay (Bio-Rad). The supernatants were then separated by SDS-PAGE (4–22.5%) in duplicate. After electrophoresis, ABCA1 was detected by an anti-ABCA1 antibody. GAPDH was used as loading control. E. Quantification of FC and CE in foam cells after interaction with CS-6253 and apoA-I. After a period of 4h cholesterol efflux, THP-1 foam cell lipids were extracted with hexane: isopropanol (3v/2v) as under ‘‘Material and Methods’. 3[H]-FC and 3[H]-CE were separated by TLC and located by exposure to iodine vapor, and were scraped off into liquid scintillating vials and assayed for radioactivity. Results are from a single experiment using triplicate wells and the mean (±SD) is presented. P<0.0001 versus control sample untreated THP-1 cells for cholesterol efflux. P<0.05 versus FC or CE in untreated cells, by Student’s t-test.

Previous studies indicated that apolipoprotein peptides can act as ABCA1-dependent acceptors for cellular cholesterol efflux [7–9, 32]. At high concentrations, other peptides displayed detergent-like properties and could extract cholesterol from cells independently of ABCA1 [33]. Notably, Remaley *et al*. demonstrated that apolipoprotein peptides composed of D-amino acids (10μg/ml) can extract cholesterol in ABCA1-dependent and independent fashions [8]. This suggests that these peptides may cause non-specific side effects compared to apo A-I [34]. To address this issue we tested our peptide at a concentration of 30μg/ml (9.69 μM) in a time- dependent manner to ensure that cholesterol efflux was ABCA1-dependent (**Fig 2A)**. Mock-transfected BHK cells and non-stimulated BHK-ABCA1 cells were used as controls. CS-6253-mediated cholesterol efflux reached saturation rapidly after ~ 6 h incubation period. In contrast, apo A-I-mediated cholesterol efflux continues to increase (**Fig 2A**). There was no significant cholesterol efflux to CS-6253 from control cells similarly to apo A-I.

### CS-6253 mediates cholesterol and phospholipid efflux via ABCA1 in a dose dependent manner

Lipid efflux was performed by the addition of lipid free apo A-I or CS-6253 at increasing concentration (0–40 μg/ml) in cAMP-treated J774 mouse macrophages (**S1 Fig**) and BHK cells expressing ABCA1 under mifepristone (**Fig 2B**). As CS-6253 was designed from the carboxyl terminal sequence of apo E we included lipid free apo E as another control for cholesterol efflux via BHK cells expressing ABCA1. Our result shows that the kinetic efficiency molar ratio of CS-6253 was ~three to five times less effective at promoting ABCA1-mediated cholesterol in BHK-ABCA1 when compared to apo E and apo A-I, respectively. Interestingly CS-6253 is two times more efficient than AT-5261. *V*_*max*_ values are increased in the BHK-ABCA1 expressing cells relative to J774 cells. Kinetic efficiencies of cholesterol efflux were determined from the *V*_*max*_ and *K*_*m*_ values expressed as (*V*_*max*_/*K*_*m*_), i.e catalytic efficiencies of CS-6253 to interact with lipids and promote ABCA1-mediated lipid efflux [27, 29]. The kinetic efficiency (*V*_*max*_ */K*_*m*_) of CS-6253 in promoting ABCA1-mediated cholesterol efflux from J774 macrophages and BHK-ABCA1 cells is higher for CS-6253 4 to 5 fold than apo A-I (**S1 Fig and Fig 2B)**. In addition, phospholipid efflux was found to be similar for CS-6253 and ATI-5261, albeit with a *K*_*m*_ of (0.33±0.05 μM) for CS-6253 vs *K*_*m*_ of (0.14±0.02 μM) for apo A-I (**S2 Fig**). Analogous to apo A-I the *V*_*max*_ of the peptide was found to promote greater cholesterol efflux in BHK-ABCA1 cells than in J774 cells (p<0.05).

### CS-6253 stimulate cholesterol efflux from human THP-1 foam cells

Over 24h human apoA-1 and CS-6253 respectively removed respectively (27.42±1.04, *P*<0.001) (25.33±0.04%, *P*<0.0001), of ^3^[H]-FC from FC-enriched THP-1 cells, compared with control (cells incubated without acceptor particles) (**Fig 2C**). Expression of ABCA1 in THP-1 macrophages-derived foam cells is confirmed by Western blot (**Fig 2D**). After 72h incubation with AcLDL and ^3^[H]-FC, cells accumulated more FC than CE as indicated by the specific activity (cpm/mg cell protein) (**Fig 2E**).

### ABCA1 oligomerization is not altered by CS-6253

We examined the oligomeric status of ABCA1 transporter in BHK cells stably expressing ABCA1 when incubated with CS-6253 by using *n*-dodecylmaltoside combined with denaturing gel electrophoresis SDS-PAGE (4–22.5%). To assess CS-6253 effects on oligomeric forms of ABCA1 compared to apo A-I, the chemical cross linker DSP was applied to BHK-ABCA1 cells [15]. As shown in (**S3 Fig**), detection of cross-linked ABCA1 by anti-ABCA1 antibody after separation of total cell lysate solubilized by a non-ionic detergent *n*-dodecylmaltoside (0.5%) on denaturing gel (4–22.5%), revealed that ABCA1 migrated primarily as monomers (~ 250kDa) or as dimers (~ 500kDa). On the other hand, using DTT as a reducing agent, we observed that all the oligomeric forms were reduced to the monomeric form with a molecular mass of <250 kDa indicative of disulfide bond contribution in dimer formation as estimated by using SDS-PAGE [25]. Densitometric quantification of the immunoblots showed a ratio of ABCA1 dimer: monomer for apo A-I of (69.22%±0.25), and CS-6253 of (56.47%±0.63, p<0.05). This data demonstrates that the peptide does not disrupt oligomerization. To rule-out the possibility that observed ABCA1 oligomerization may be the result of either nonspecific protein aggregation or the formation of nonspecific disulfide linkages during cell lysis and membrane preparation, urea 4 M and 100 mM iodoacetate were used. The use of urea in SDS-PAGE did not alter the ABCA1 dimer-monomer ratio, suggesting that ABCA1 dimerization is not the result of nonspecific aggregation of ABCA1 protein in our assay. Also, the addition of iodoacetate, a cysteine reactant known to prevent the formation of di-sulfide bonds, to the homogenization medium failed to prevent formation of ABCA1 dimers (data not shown). Thus CS-6253 peptide like apo A-I causes ABCA1 dimer formation.

### CS-6253 competes as effectively as apo E with apo A-I for binding to ABCA1

**Fig 3:**
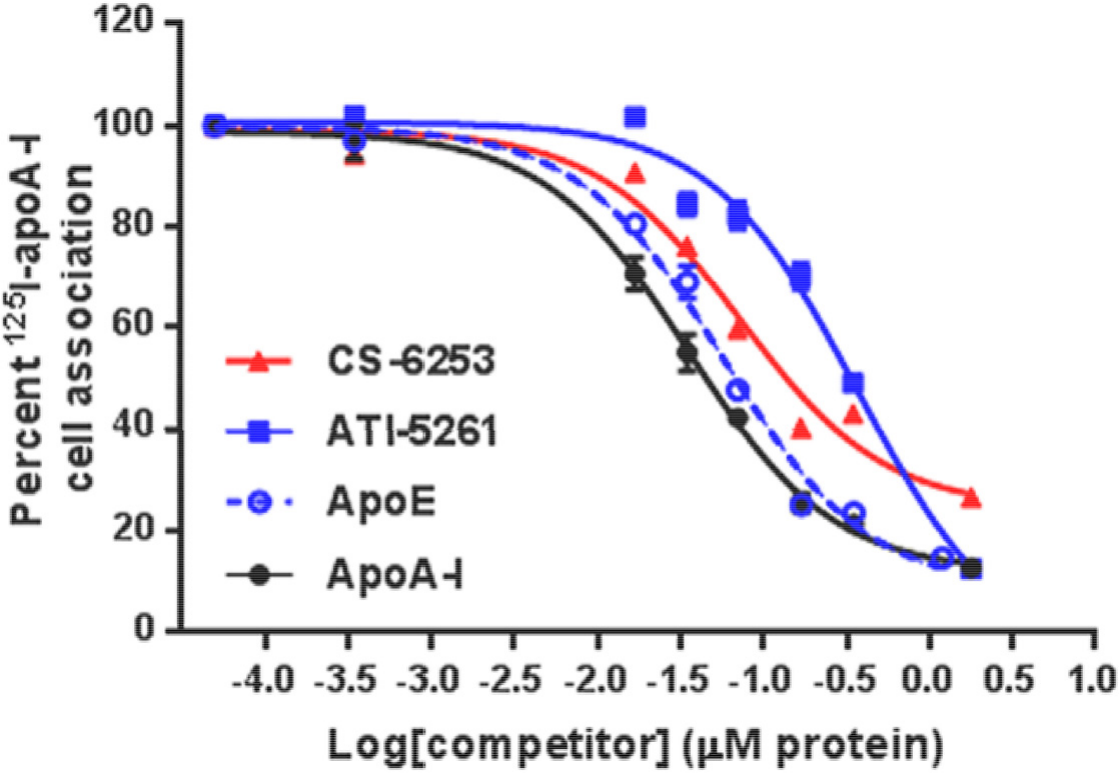
Competitive binding of CS-6253 peptide to ABCA1 cells expressing ABCA1. BHK-ABCA1 cells were plated in 24-well plates and stimulated for 20 h. Cells were then incubated with 2 μg/ml of ^125^I-apo A-I for 2 h at 37°C with increasing molar concentrations relative to apo A-I of either CS-6253 peptide, ATI-5261, human apo E, and unlabelled apo A-I (0, 0.0035, 0.017, 0.035, 0.071, 0.17, 0.35, 1.79 μM). Cells were then washed rapidly three times with ice-cold PBS/BSA and then PBS alone. ^125^I-apo A-I cell associated was determined as described under “Experimental Procedures.” The values shown represent the mean ± S.D from triplicate wells. The 100% of control value measured in the absence of competitors was 0.21 ng of apo A-I /μg cell protein. Values of IC_50_ shown were determined using the Graph Pad Prism 6 software.

Competition studies were performed to determine the ability of CS-6253 to compete for the binding of ^125^I-apo A-I to ABCA1 in stimulated BHK-ABCA1. Lipid free CS-6253 inhibited the binding of ^125^I-apo A-I to ABCA1 as efficiently as apo A-I and apo E, i.e. (IC_50_ = 0.035±1.66 μM) for apo A-I, (0.057±1.53 μM) for apo E and (0.072±1.41 μM) for CS-6253 (**Fig 3**). Control experiments were conducted to examine whether the apparent decrease in cell binding of the labeled apo A-I may be attributable to the ^125^I-apo A-I binding to different competitor particles instead of the cells, as described in experimental procedures (data not shown). No significant amount of ^125^I-apo A-I was found associated with CS-6253, supporting the results shown in **Fig 3**. Control data generating IC_50_ were performed in the same experiment for CS-6253 and ATI-5261, as previously published (7).

### CS-6253 promotes nascent (n) HDL formation through desorption of specific lipids from plasma membrane micro-domains

**Fig 4:**
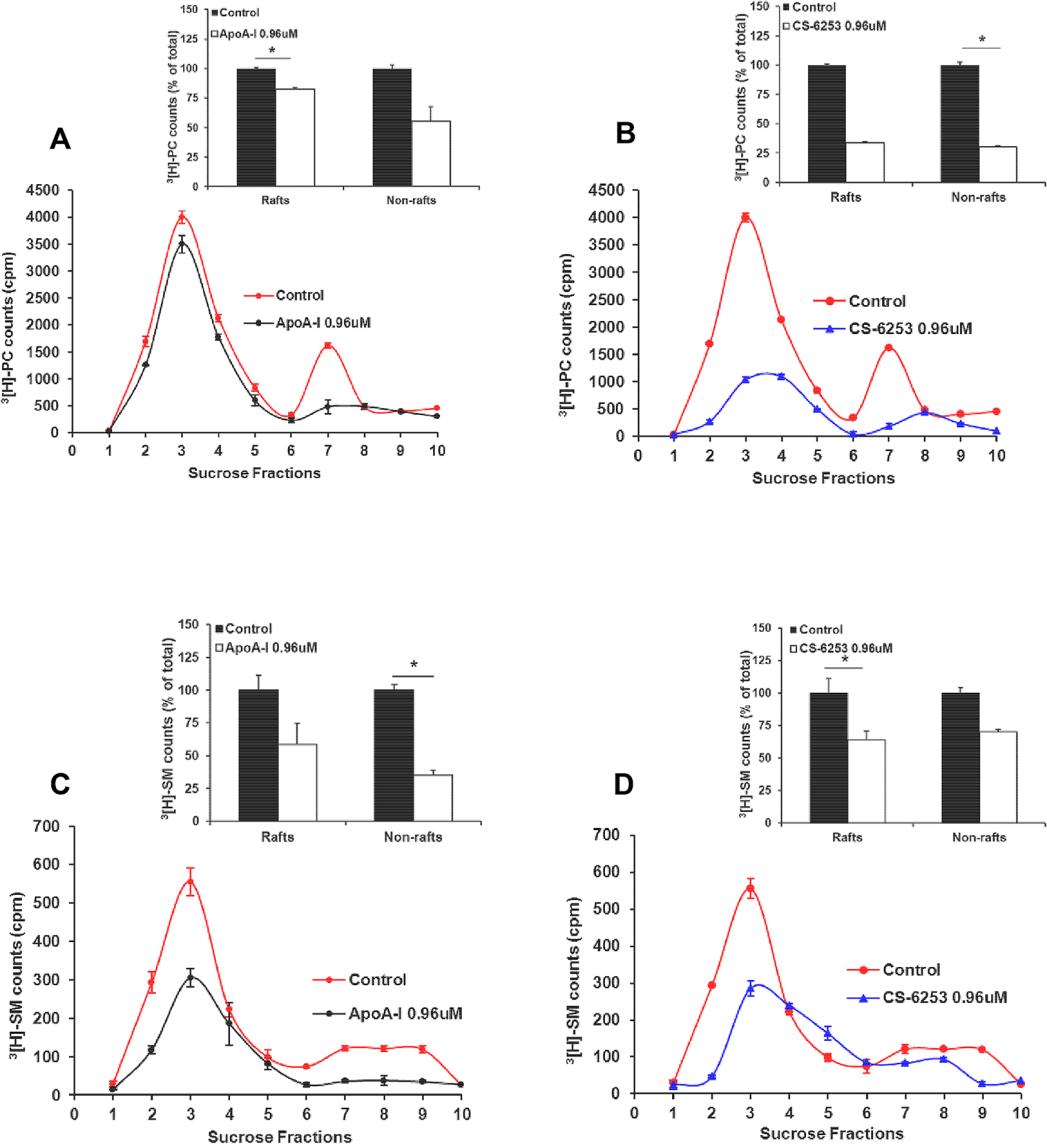
Apo A-I (left panels) and CS-6253 (right panels) desorb phospholipids species from raft and nonraft domains. BHK-ABCA1 cells were labeled with ^3^[H]choline for 48 h, followed by stimulation with mifepristone for 18–20h as described in “experimental procedures”. Cells were then incubated for 12h with apo A-I or CS-6253 (0.96 μM). After lipid extraction by Folch, ^3^[H]phosphatidyl choline (^3^[H]PC) (A and B), or ^3^[H]sphingomyelin (^3^[H]SM) (C and D) in each fraction was assessed for radioactivity. Radioactivity appearing in fractions corresponding to raft (1–5) and nonraft (6–10) material was pooled, and desorption of ^3^[H]PC (A and B inset) and ^3^[H]SM (C and D inset), from raft versus nonraft in the presence of apo A-I or CS-6253 was expressed as a percentage of control (100%, in the absence of acceptor). ^3^[H]PC and ^3^[H]SM distributions in sucrose gradient fractions after 12h incubation with apo A-I or CS-6253 were assessed by TLC respectively. Results shown are representative of three independent experiments. *P < 0.05 by Student's t-test.

We investigated the role of PM lipid microdomains in the formation of nHDL-CS-6253 at the cellular level. The PM levels of cholesterol and total phospholipids separated by sucrose fractionation were measured by spectroscopy. Addition of apo A-I or CS-6253 for 45 min, 6 h or 12 h to BHK-ABCA1 cells promotes significant lipid desorption from both raft and non-raft domains (**S4 and S7 Figs**). Incubation with CS-6253 was found to promote significantly cholesterol desorption from both rafts and non-rafts after 45 min respectively (20% CS-6253 vs 25% apo A-I) (**S4 and S5 Figs,** *inset*) and only from rafts after 6 h incubation (data not shown). No cholesterol removal was seen following 12 h incubation with CS-6253 in contrary to apo A-I (data not shown). Phospholipids desorption after 45 min incubation was similar for both apo A-I and CS-6253 (**S6 and S7 Figs**). Moreover, we sought to investigate the effect of CS-6253 (0.96 μM) on PM phospholipids species, i.e phosphatidylcholine (PC) and sphingomyelin (SM) after 12h incubation with cells and using apo A-I as control [35]. Incubation with apo A-I (0.96 μM) was found to promote less PC desorption in PM microdomains than CS-6253 with 18% significant reduction in raft fraction vs. control (p<0.05) (**Fig 4A and 4B**). In addition, SM were found to be desorbed from both non-raft and raft domains as shown by apo A-I and CS-6253 profile distributions (**Fig 4C and 4D**). This is consistent with quantification of radioactivity appearing in rafts (fractions 1 to 5) and non-rafts (fractions 6 to 10) expressed as percentage of control (100%, in the absence of apo A-I) for ^3^[H]PC and ^3^[H]SM (**Fig 4A, 4B, 4C and 4D** *inset*).

### Nascent HDL particles are generated by CS-6253 through ABCA1

**Fig 5:**
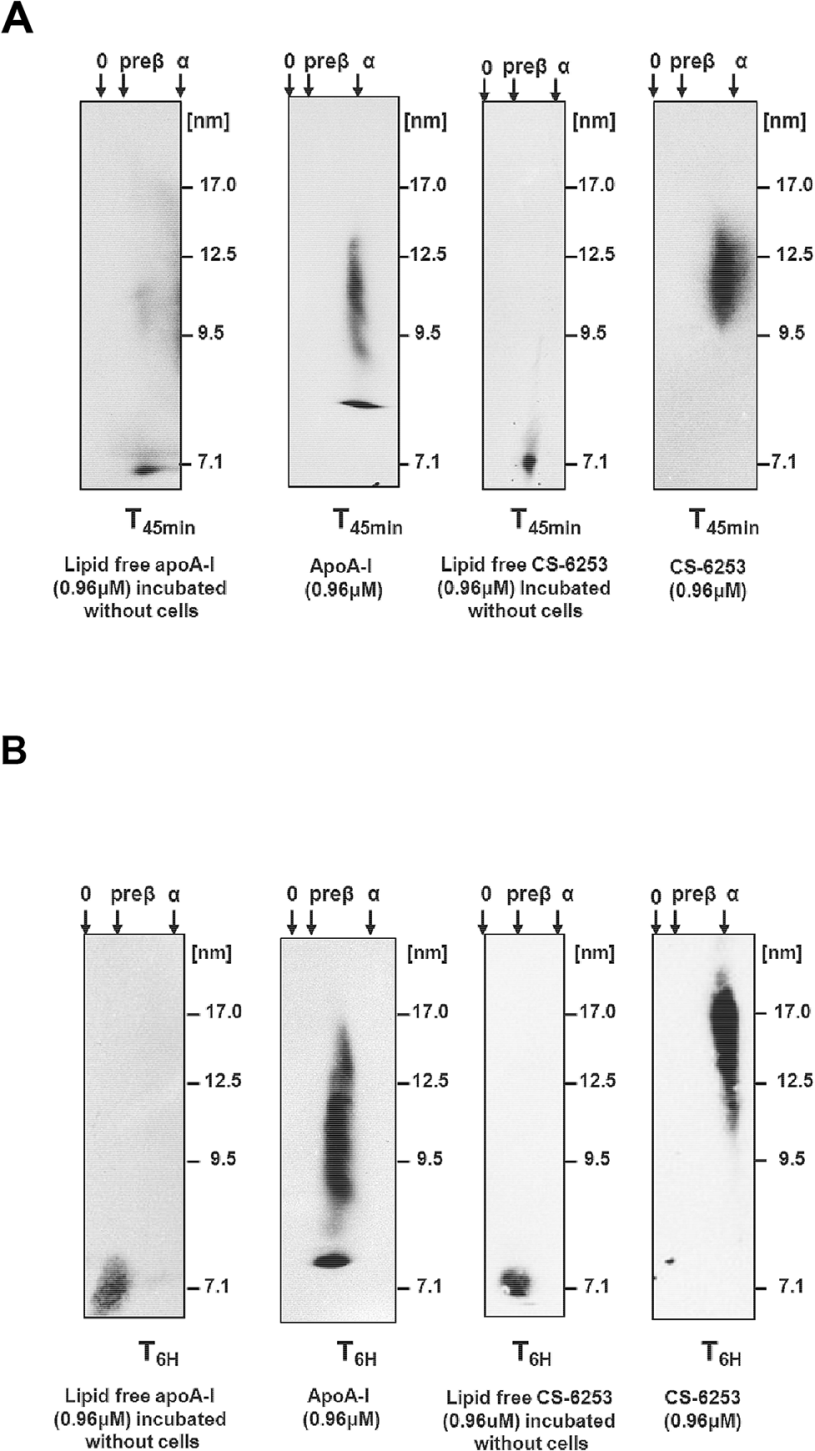
Time course ability of CS-6253 to generate nHDL-mimetic like particles. (A and B): nHDL-CS-6253 and nHDL-apo A-I released to the medium at specific time points (45 min and 6 h) from BHK cells expressing ABCA1 were analyzed by 2D-PAGGE. Apo A-I was detected by polyclonal anti-apo A-I, CS-6253 peptides was detected by anti-CS-6253 and revealed by chemiluminescence. Molecular markers are indicated.

Next, we investigated the nature of CS-6253-containing particles released in the medium from stimulated ABCA1 cells. Cells were first incubated in 100 mm diameter dishes with either (0.96 μM) apo A-I or CS-6253 in 8 ml of DMEM for 45 min and 6 h at 37°C. These two time points were selected based on our previous work on HDL biogenesis [25, 36]. Lipid free apo A-I or CS-6253 incubated in cell free system media under same conditions were used as control for 2D-PAGGE analysis. Apo A-I or CS-6253-containing particles were separated and analyzed by 2D-PAGGE. As shown in **Fig 5**, left panel, apo A-I or CS-6253 incubated without cells had a pre-β electrophoretic mobility with a molecular diameter of 7.1 nm. However, apo A-I and CS-6253-containing particles released from stimulated ABCA1 cells at either 45min or 6 h exhibited α-electrophoretic mobility with a particle size ranging from 9 to 20 nm (designated α-LpA-I-like particles, α-LpCS-6253-like particles) (**Fig 5A and 5B**) respectively. Both the charge and size of these nascent particles were stable over a 6h incubation period. These nHDL particles were retained following 50 kDa molecular weight cut filter separation, suggesting a particle size that is equal to, or greater than 20 nm nHDL-LpA-I [21].

### nHDL-CS-6253 promote ABCG1-mediated cholesterol efflux in a time- and concentration- dependent manner

Cells expressing ABCA1 were incubated separately with 0.96 μM lipid free apo A-I or CS-6253 for 18h as previously reported [13]. The conditioned medium was removed and added to ^3^[H]cholesterol-labeled BHK cells expressing ABCG1 in time dependent manner (0–6h) [27]. ABCG1-mediated cholesterol efflux was significantly higher for nHDL-apoA-I compared to nHDL-CS-6253 after 6h incubation (**S8 Fig**). In another experiment we used apo A-I and CS-6253 conditioned medium from 18 h of incubation with unlabeled BHK cells expressing ABCA1 as previously reported [12, 13, 37]. Increasing concentrations of lipid free or nHDL using native apo A-I or CS-6253 was added to ^3^[H]cholesterol-labeled ABCG1 for 6 h, and assayed for cholesterol efflux (**S9 Fig**). Kinetic efficiency of nHDL-apo A-I was eight times more efficient in promoting ABCG1-mediated cholesterol than nHDL-CS-6253. When lipid free CS-6253 was directly incubated with BHK-expressing ABCG1, ^3^[H]cholesterol efflux was linear over the dose range and was markedly lower than that of lipid free apo A-I (**S9 Fig)**. The ^3^[H]cholesterol released from ABCG1 was saturable and had a lower *K*_*m*_ molar efficiency for the lipid free CS-6253 peptide (0.18±0.15 μM) when compared with apo A-I (0.06 ± 0.01 μM)). Thus, lipid free CS-6253 peptide is a relatively poor acceptor for ABCG1 cholesterol efflux. Interestingly the CS-6253 in lipid free form is a poor acceptor that still retained some net cholesterol efflux capacity through ABCG1 reaching maximum of less than 0.5% above the top of the base line vs 1% by apo A-I (**S9 Fig)**.

### nHDL mimetic CS-6253 particles are remodeled in plasma

We further examined the effect of nHDL generated with apo A-I or CS-6253 on LCAT and PLTP activities in plasma. LCAT activity was determined by the fractional esterification rate (FER) in the presence of apo A-I or CS-6253 with, or without the LCAT inhibitor DTNB as described in Experimental procedures. As shown in **S10 Fig**, both apo A-I and CS-6253 increased LCAT activity, similar to nHDL-apo A-I (FER = 7.74±0.35%/h; vs nHDL CS-6253 FER = 7.41±0.25%/h). The presence of DTNB resulted in the inhibition of LCAT by (98.37 ± 0.08%). Two dimensional PAGE analysis confirmed the formation of larger (12~17 nm) HDL particles after incubation with human plasma (data not shown). We then looked at phospholipid transfer between nHDL-CS-6253 and apoB containing lipoproteins in human plasma. Incubation of nHDL-CS-6253 with normolipidemic plasma in the presence of the PLTP stimulator AEBSF resulted in the transfer of a significant proportion of the ^3^[H]choline from nHDL-sized particles to apoB lipoproteins (**S11 Fig**). AEBSF increased PLTP activity by (34.6±0.72%) in nHDL-apo A-I vs (17.41±0.21%, p≤0.05) in nHDL-CS-6253 containing plasma when compared with untreated plasma. Consistently, CS-6253 nHDL increased PLTP activity in plasma by 10% relative to apo A-I (**S11 Fig**).

### FPLC cholesterol profiles of CS-6253 in media cell culture

**Fig 6:**
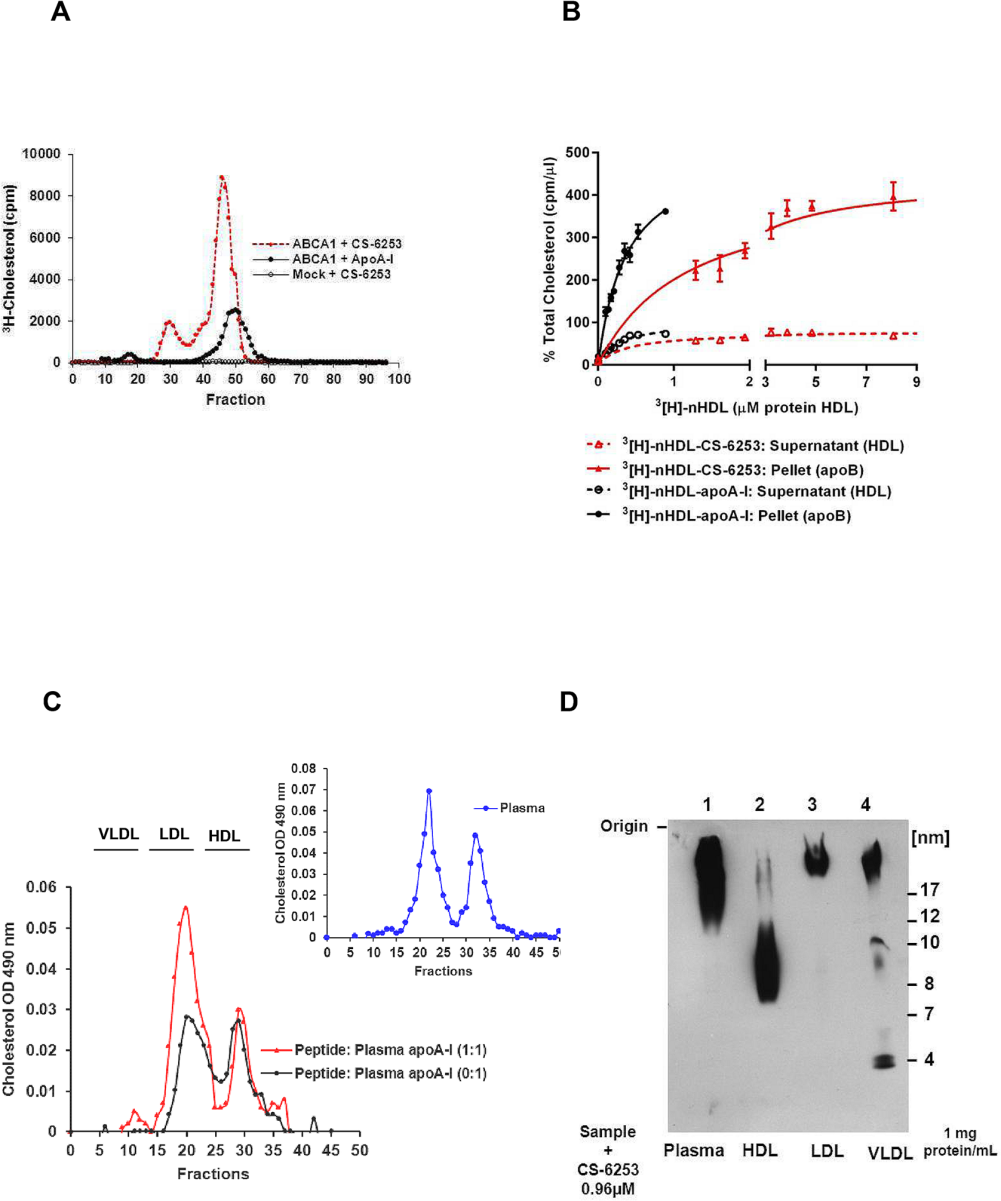
Dynamics of transfer and redistribution of BHK-ABCA1 cell-derived cholesterol content of nHDL-CS-6253. nHDL-CS-6253 particles were labelled with cell derived ^3^[H]cholesterol and incubated with normolipidemic plasma for various time periods at 37°C (1μg peptide: 10μg plasma apo A-I). A. Characterization of lipid particles released to the medium in the presence of CS-6253. ^3^[H]-cholesterol-labelled BHK cells expressing ABCA1 or not were incubated in the presence of 0.96 μM apo A-I (solid line) or CS-6253 (dashed line) for 45min at 37°C. Concentrated medium from cells was analysed by FPLC, and radioactivity associated with each fraction was determined. B. Transfer of total ^3^[H]cholesterol from ^3^[H]nHDL-CS-6253 to plasma lipoproteins. nHDL-CS-6253 or nHDL-apo A-I were generated after incubation (4h) of BHK cells expressing ABCA1 with increased doses of lipid free CS-6253 or apo A-I. Media cell culture containing ^3^[H]-nHDL-particles was incubated in human plasma for 1h, 37°C. After ApoB precipitation, fractions were dialysed and counted for radioactivity. Kinetic parameters for cholesterol transfer to apoB particles in plasma: nHDL-CS-6253 (439±0.20μM), *V_max_* (439± 19.74%cpm/μl) vs nHDL-apo A-I (0.37 ± 0.035), *V_max_* (517±24.45).C. Effects of *ex vivo* incubation of CS-6253 on plasma cholesterol lipoproteins. FPLC cholesterol profiles (OD 490 nm) of plasma apo A-I: peptide ratio (1:1) (dashed line) and plasma apo A-I: peptide ratios (1:0; i.e. apo A-I alone) (solid line) are obtained after 5 min incubation at 37°C. In all experiment counts were made in triplicate, (*P<0.05 by Student‘s t-test). For reference purposes, the *inset* shows an FPLC profile of normal plasma. D. Effect of *ex vivo* incubation of CS-6253 on plasma lipoproteins after separation by ND-PAGGE (5–35%) electrophoresis. CS-6253 (0.96 μM final dose) was incubated for 1h at 37°C with HDL, LDL or VLDL isolated by ultracentrifugation or with human plasma and then separated by electrophoresis at concentration of 1 mg/protein/mL. Gel is followed by Western blot analysis with anti-CS-6253 antibody. Internal control was run on the left of the gel and detected by Ponceau S.

To characterize cholesterol content in particles released from BHK cells expressing ABCA1 in media and plasma; lipoproteins profiles were determined by FPLC. After radiolabelling cells with ^3^[H]cholesterol for 24 hours, efflux medium (45 min) was collected, concentrated and analyzed. The elution profiles of ^3^[H]cholesterol are shown in (**Fig 6A**). In the presence of CS-6253, BHK cells produced a larger peak than apo A-I (fractions: 45–55) (**Fig 6A**), associated with an increase in the size of nHDL-CS-6253. nHDL-CS-6253 was confirmed by Western blot (data not shown) similar to nHDL-apo A-I [38].

### *Ex-vivo* effect of CS-6253 on plasma lipoproteins

In a separate experiment, transfer of cholesterol to apoB lipoproteins was examined by counting radioactivity in PEG precipitated plasma fractions (pellet and supernatant) following incubations with increased doses of radiolabelled nHDL-CS-6253 vs nHDL-apoA-I (1h, 37°C) (**Fig 6B**) derived by ABCA1 interaction. Data shows that CS-6253 promotes efficient cholesterol transfer to apoB particles in plasma with higher *V*_max_ compared to apo A-I. Treatment of plasma with equimolar ratios of CS-6253 and apo A-I for 5min at 37°C, showed that the former was associated with a larger increase in the cholesterol content in the LDL particles albeit size differences by FPLC were not observed (**Fig 6C**). A relative radiolabel increase was observed in the VLDL>LDL>HDL for peptide when normalized to plasma (data not shown). The *ex-vivo* effect of CS-6263 treatment on isolated HDL, LDL and VLDL and plasma lipoproteins was also examined after separation by ND-PAGGE and CS-6253 Western blots detection (**Fig 6D**). When CS-6253 was added to plasma (**Fig 6D,** lane 1), it increased the intensity of the band in the LDL/VLDL region. CS-6253 was found to form a major band in the (α) position (7–12nm) when incubated with HDL (**Fig 6D**, lane 2). When CS-6253 was incubated with isolated LDL (**Fig 6D**, lane 3), the band was superimposed on LDL, with no band in the pre-β region (~4nm). Finally, when CS-6253 was added to isolated VLDL (**Fig 6D**, lane 4), it shows an increased band in the VLDL position, leaving a residual band in the pre-β region (lipid free CS-6253)

### Addition of lipid free CS-6253 or nHDL-CS-6253 to human plasma stimulates ABCA1-dependent cholesterol efflux

**Fig 7:**
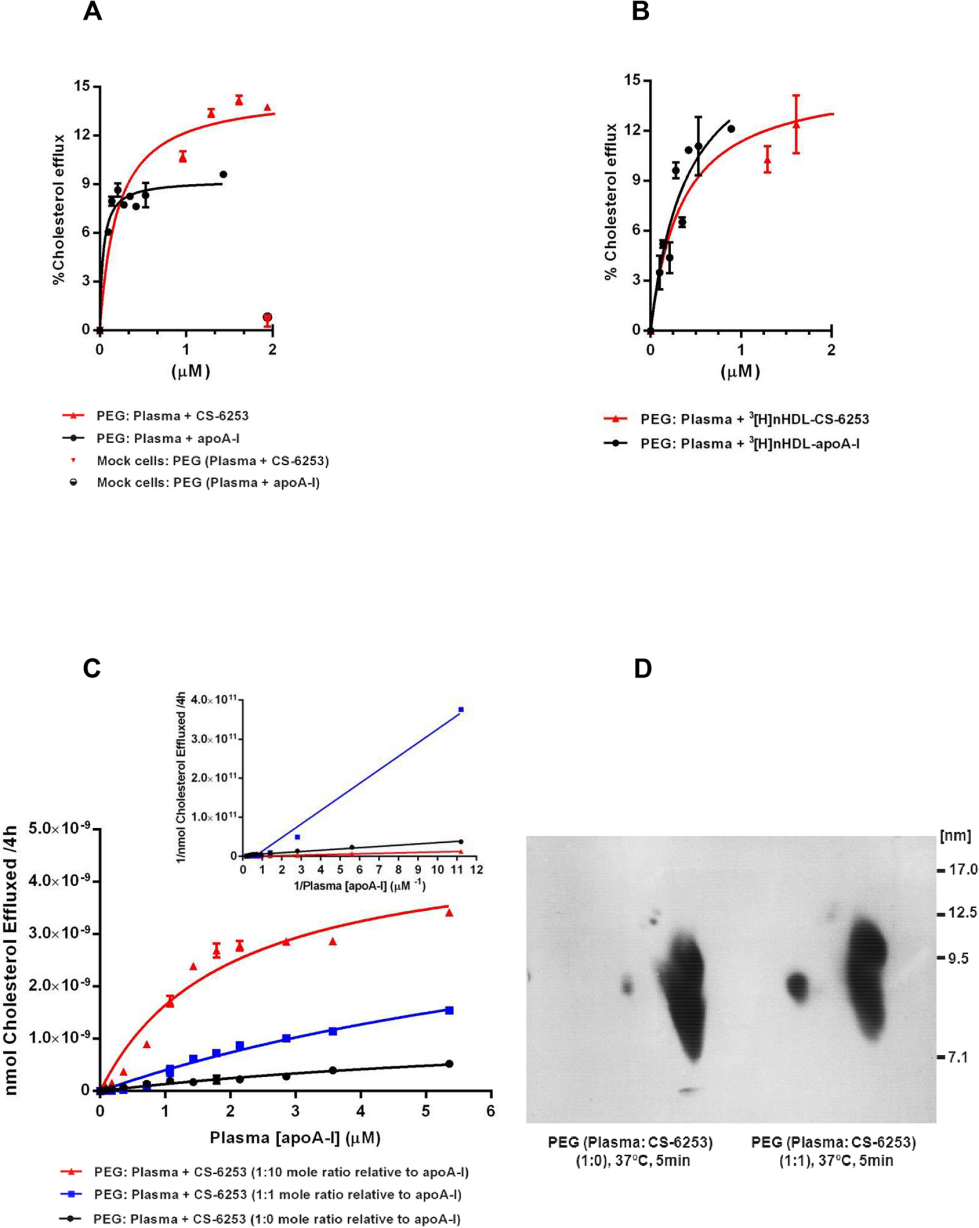
Dose dependent effects of pre-incubation of plasma with lipid free CS-6253, or nHDL-CS-6253 on ABCA1 cholesterol efflux. A. Increased doses of lipid free CS-6253 or apo A-I were added to normolipidemic plasma and incubated for 1h at 37°C. A volume of 2.8% of HDL fractions isolated by PEG was used for the measurements of cholesterol efflux from BHK cells expressing ABCA1 and mock cells. Kinetic parameters for ABCA1-mediated cholesterol efflux from BHK expressing ABCA1 cells to apo A-I in plasma: *K_m_* = 0.03±0.008 μM, *V_max_* = 9.26±0.29% efflux/4h. CS-6253: *K_m_* = 0.20±0.05 μM, *V_max_* = 14.71±0.39% efflux/4h. Kinetic parameters for cholesterol efflux from BHK-mock cells to apo A-I in plasma: *K_m_* = 0.07±0.95 μM, *V_max_* = 0.30±0.15% efflux/6h. CS-6253: *K_m_* = 0.65±0.63 μM, *V_max_* = 0.84±0.17% efflux/4h. B. nHDL-CS-6253 and nHDL-apo A-I were generated as indicated above. nHDL particles were incubated in plasma for 1h at 37°C, and specific HDL fractions isolated by PEG were subjected to cholesterol efflux in ^3^[H]-radiolabelled BHK cells. Kinetic parameters for ABCA1-mediated cholesterol efflux from BHK expressing ABCA1 cells to nHDL-apo A-I in plasma: *K_m_* = 0.25±0.08 μM, *V_max_* = 15.73±2.14% efflux/4h. nHDL-CS-6253: *K_m_* = 0.32±0.15 μM, *V_max_* = 15.01±0.84% efflux/4h. C. Mobilization of cholesterol efflux of increasing concentrations of peptide: plasma apo A-I molar ratios: (1:0) (closed circles), (1:10) (upright closed triangles), and (1:1) (closed squares). (C *inset*). The double reciprocal-plot was used to calculate the apparent appK_*m*_ of the efflux activity in peptide: plasma with indicated ratios. Kinetics values are as follows, molar ratio of plasma apo A-I: CS-6253 (1:0); app*K_m_* (28±0.07 μM). Molar ratio of plasma apo A-I: CS-6253 (1:1); app*K_m_* (4.17±0.47 μM). Molar ratio of plasma apo A-I: CS-6253 (1:10); app*K_m_* (0.71±0.46 μM). D. Lipid free CS-6253 was incubated with normolipidemic plasma, using peptide: apo A-I mole ratio of (1:1) for 5 min at 37°C versus plasma alone. Isolated HDL fractions by PEG were separated by 2D-PAGGE and HDL subpopulations were detected by apo A-I Western blot. Molecular size markers are shown. In all experiments, efflux of ^3^[H]cholesterol is shown as means ± SD of triplicate experiments.

Plasma cholesterol efflux from BHK-cell following increased doses of lipid free CS-6253 was assessed and compared with CS-6253 alone (media DMEM). The efflux capacity of plasma-CS-6253 in BHK-mock cells reached a maximum of less than 1% similar to apo A-I in plasma (**Fig 7A**). The ABCA1-mediated efflux by lipid free CS-6253 in plasma was four times more efficient than that caused by the peptide alone in media (**Table 1**). Increased doses of nHDL-CS-6253 or nHDL-apo A-I were generated as indicated above. The ABCA1-mediated efflux by nHDL-CS-6253 in plasma was two times less efficient than that caused by the peptide in plasma (**Fig 7B**) (**Table 1**). Analogous with lipid free apo A-I the peptide generated more efficient cholesterol efflux in plasma than that found in media (**Table 1**). The data shows that CS-6253 mediates ABCA1 dependent efflux in plasma. Furthermore, CS-6253 efflux in plasma is two times more efficient than nHDL-CS-6253 (**Table 1**). This was associated with higher *V*_*max*_ of the ABCA1 efflux in plasma with CS-6253 than with apo A-I (**Table 1**). To better define the effect of lipid free CS-6253 on plasma cholesterol efflux capacity, increasing doses of CS-6253 were added to human plasma with molar ratios of (0:1), (1:1) and (10:1) peptide: plasma apo A-I for 5min at 37°C. HDL specific cholesterol efflux (4h) was then evaluated using BHK cells expressing ABCA1. To analyze kinetics of peptide, ABCA1 cholesterol efflux was expressed in nanomoles cholesterol efflux (**Fig 7C**). Values obtained were used to generate a Lineweaver-Burk double reciprocal plot to determine apparent *K*_*m*_ (app*K*_*m*_) (**Fig 7C,** *inset*). The app*K*_*m*_ values were significantly different for (28±0.072 μM) plasma apo A-I alone vs. (4.17±0.47 μM) for CS-6253 with plasma-apo A-I (1:1), and (0.71±0.46) for (10:1) ratio, p<0.0001). The app*K*_*m*_ for plasma alone demonstrate lower potency to activate efficient cholesterol efflux than with CS-6253 added.

**Table 1:** Comparison of kinetic parameters for ABCA1-mediated cholesterol efflux (4h) from BHK cells in media cell culture and human plasma. Data are presented as mean ± SD.

Acceptor	Efflux condition	*K*_*m*_ (μM)	*V*_*max*_ (% efflux/4h)
**Lipid free CS-6253**	**media**	**0.73±0.05**	**15.25±0.23**
**Lipid free ATI-5261**	**media**	**0.37 ± 0.04**	**14.48 ±0.29**
**Lipid free apo A-I**	**media**	**0.15±0.02**	**14.85±0.69**
**nHDL-CS-6253**	**plasma**	**0.34±0.16**	**15.28±0.93**
**nHDL-apo A-I**	**plasma**	**0.40±0.11**	**18.45±2.48**
**Lipid free CS-6253**	**plasma**	**0.19±0.04**	**14.60±0.31**
**Lipid free apo A-I**	**plasma**	**0.02±0.0091**	**8.81±0.33**

### Addition of lipid free CS-6253 to human plasma induces pre β1-HDL formation via mechanisms involving distinct α-HDL subpopulations

**Fig 8:**
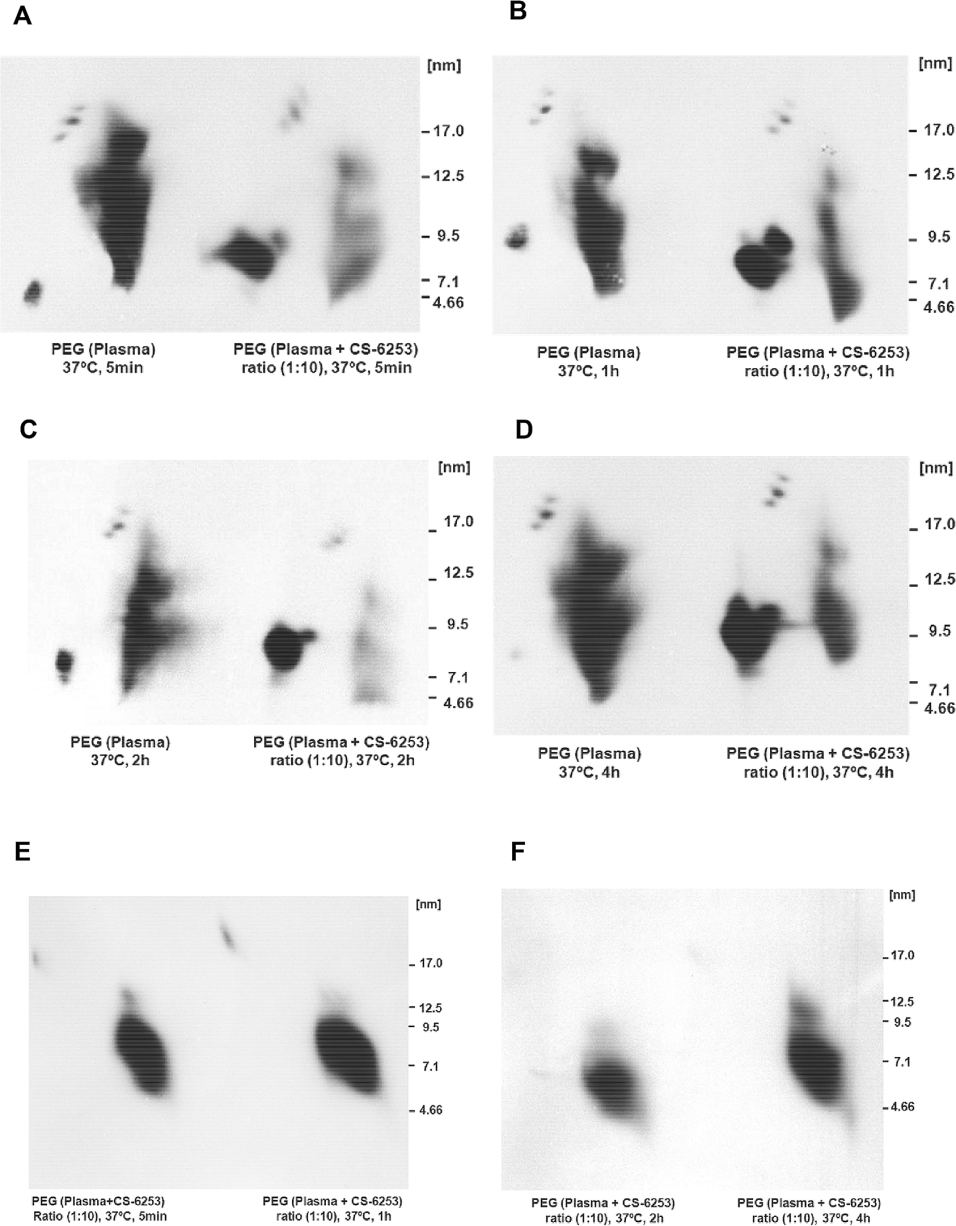
Time dependent effects of incubation of lipid free CS-6253 with human plasma, on HDL subparticles. (A, B, C and D). Lipid free CS-6253 was incubated with normolipidemic plasma, using peptide: apo A-I molar ratio of (10:1) and (0:1 i.e. no peptide) for (5 min, 1h, 2h, and 4h) at 37°C. HDL subpopulations were detected by an anti-apo A-I antibody. E and F Samples were then PEG precipitated to remove apoB-containing lipoproteins and separated by 2D-PAGGE. HDL subfractions were detected by an antibody against CS-6253. Molecular size markers are shown.

**Fig 9:**
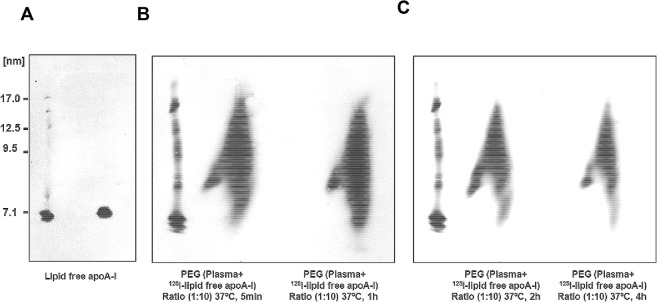
Time dependent effects of incubation of lipid free CS-6253 with human plasma, on HDL subparticles. (A, B, C). Radiolabeled Lipid free apoA-I was prepared as indicated in “Material and Methods”. ^125^I-lipid free apoA-I in incubated with normolipidemic plasma, using molar ratio (10 μg Lipid free apoA-I or peptide: 1 μg plasma apoA-I) at 37°C for increasing time for (5 min, 1h, 2h, and 4h) at 37°C. Samples were separated by 2D-PAGGE, and ^125^I-apoA-I was detected by autoradiography. Lipid-free ^125^I-apoA-I (C) incubated in PBS for 4 h at 37°C is shown as controls. Molecular size markers are shown.

Cholesterol efflux results in **Fig 7C** showed that PEG precipitated plasma with peptide at (1:1 mole ratio relative to apo A-I) increased preβ-1 HDL 4-fold (±1.12SD), as determined by densitometry analysis, when compared to non-treated plasma (**Fig 7D**). Having observed efficient increase of ABCA1 mediated cholesterol efflux to CS-6253 in plasma or in plasma molar ratios relative to apo A-I, we investigated the effects of CS-6253 on HDL particles by 2D-PAGGE (**Fig 8A, 8B, 8C and 8D**). We observed that in a time-course manner the peptide altered the size distribution of the larger HDL-apo A-I species α-1 and α-2 with ratio of (1:10) (**Fig 8A, 8B, 8C and 8D**) with near-complete conversion of α to preβ-1 HDL achieved at 5 min or 2h. This was associated with dramatic increase in preβ-1 particles, 31-fold after 5min and 26-fold after 2h (**Fig 8A and 8C**). Interestingly, we found that the CS-6253 peptide itself is unable to associate with preβ-1 HDL, and as it bound only to α–HDL species as judged by 2D-PAGGE and CS-6253 Western blots using CS-6253 specific anti-body (**Fig 8E and 8F**). CS-6253 or ^125^I-Lipid free apoA-I (control) were incubated with normolipidemic plasma ratio (10 μg Lipid free apoA-I or peptide: 1 μg plasma apoA-I) at 37°C for increasing time. When ^125^I-lipid free apoA-I was added to plasma we observed only a 0.23-fold increase in preβ-1 particles after 5min and 0.4-fold increase after 2h **(Fig 9B and 9C).**

### SR-BI mediates nHDL-CS 6253 cholesterol uptake in Fu5AH cells

**Fig 10:**
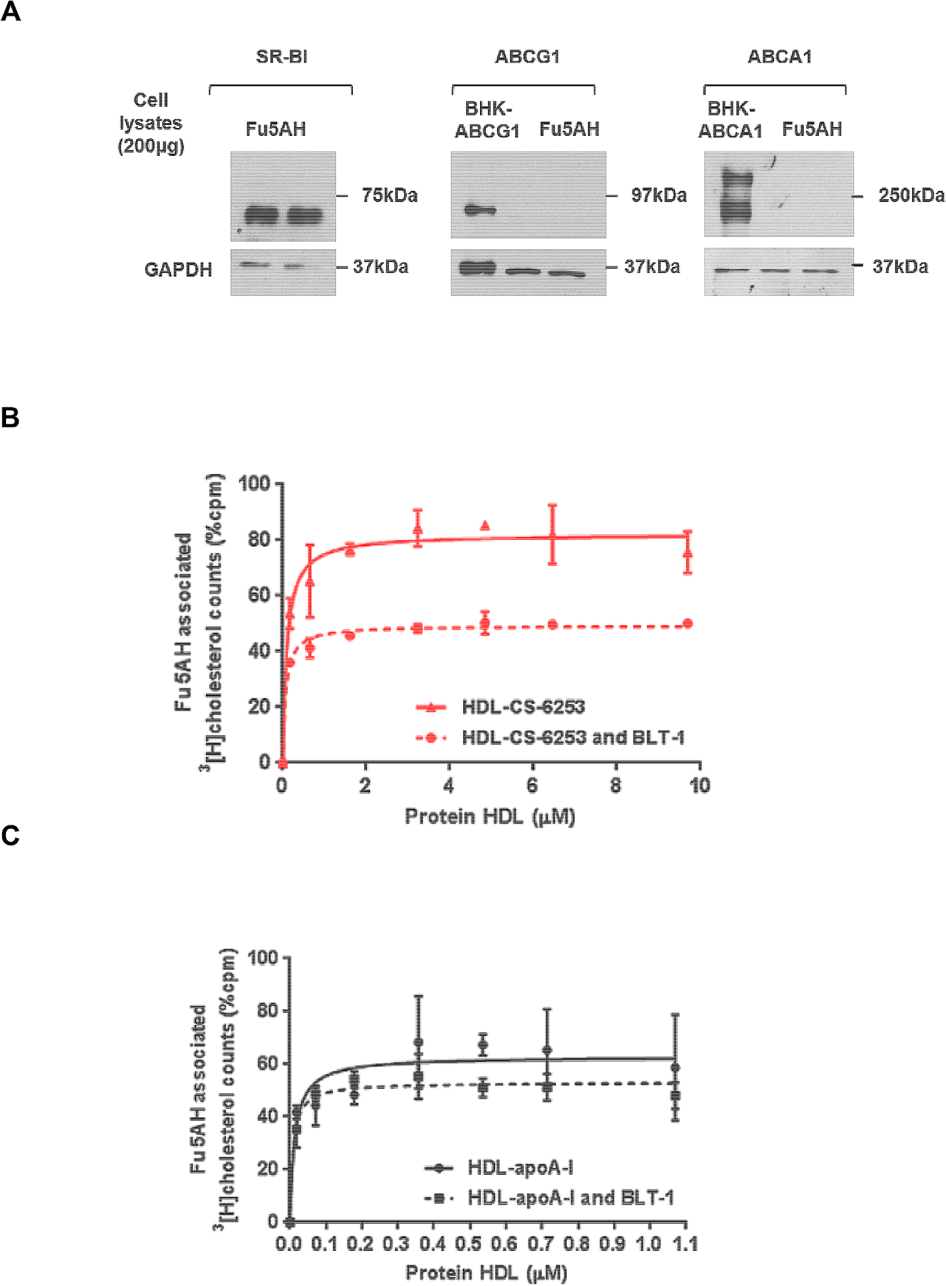
Uptake of cholesterol to hepatic cells expressing SR-BI from plasma containing HDL-CS-6253 or HDL-apo A-I. A. Fu5AH liver hepatoma cells expression of SR-BI transporter ability. Fu5AH cells were grown as described in Experimental procedures, cells were lysed at 4°C with (20mM Tris, 5mM EDTA, and 5 mM EGTA; pH 7.5) containing 0.5% *n*-dodecylmaltoside. The suspension was subjected for further lysis in the presence of protease inhibitor mixture followed by low speed centrifugation to remove insoluble material. Protein concentration was determined by standard assay (Bio-Rad). Cells were separated by SDS-PAGE (4–22.5%), and immunoblotted using antibodies against human SR-BI, ABCG1 and ABCA1. Cells lysate from BHK cells expressing ABCG1 and ABCG1 was used as positive control. Molecular weight (Bio-Rad) is shown on the left of the gel. B and C. Radiolabelled nHDL-CS-6253 or nHDL-apoA-I particles were generated after incubation of J774 cells with lipid free CS-6253 or apo A-I at increased concentrations as showed in experimental procedures. nHDL particles with CS-6253 or apoA-I were then incubated in plasma to induce lipidation. Cholesterol uptake by SR-BI Fu5AH cells was determined by incubation for 6h with 20% apoB depleted plasma as described in experimental procedures. BLT-1 was used to specifically inhibit SR-BI. The values shown represent the mean ± S.D from triplicate wells.

The uptake of cholesterol from HDL particles to hepatocytes represents the final step in the RCT pathway. We determined the SR-BI-mediated cholesterol uptake from nHDL generated with CS-6253 in Fu5AH cells that express SR-BI. ApoA-I nHDL were used as control, as previously described [27]. SR-BI was inhibited incubating the cells for 2 hours with a small molecule blocker of lipid transport (BLT-1). Cell-associated ^3^[H]cholesterol was quantified as described in Experimental procedures. Fu5AH cells do not express ABCA1 or ABCG1 as determined by Western blot analysis (**Fig 10A**). The HDL-CS-6253 particles were less efficient in promoting total cholesterol delivery to Fu5AH cells (0.11±0.02 μM) than HDL-apo A-I (0.01±0.004 μM). Interestingly, nHDL-CS6253 particles were ~3 fold more efficient in delivering cholesterol to hepatic cells through SR-BI in human plasma than nHDL-ATI-5261. BLT-1, a specific inhibitor of SR-BI was used to examine the specificity of cholesterol delivery to Fu5AH cells by CS-6253 HDL particles **(Fig 10B).** BLT-1 inhibited uptake of cholesterol from CS-6253 or apo A-I nHDL. This data confirms the selectivity of SR-BI-mediated cholesterol uptake (**Fig 10C**). Thin layer chromatography was used to measure cholesteryl ester composition associated with these cholesterol delivering particles and apo A-I nHDL was found to have significantly more CE than CS-6253 nHDL (2497±84 cpm versus 1784±19 cpm, p<0.05)

## Discussion

In the present study, we analyzed effects of the apolipoprotein-mimetic peptide CS-6253, compared with apo A-I on HDL functions across sequential key steps in the RCT pathway. The ABCA1 transport is rate-limiting in HDL formation [39] and therefore potentially a therapeutic modality of interest [7, 9, 40, 41].

We first studied HDL lipid efflux in BHK cells stably expressing the ABCA1 transporter, in a time and dose response manner. CS-6253 was dependent on ABCA1 for stimulating cellular cholesterol efflux in a manner analogous to native apo A-I (**Fig 2A**). Cholesterol efflux by diffusion to apo A-I or CS-6253 was examined in BHK mock cells not expressing ABCA1, and in unstimulated BHK-ABCA1 cells. Both were similar, reaching a maximum of 1% for CS-6253 vs 0.75% for apo A-I. This finding is in contrast to that observed with synthetic peptide 37pA, which effluxes cholesterol from cells using ABCA1-dependent and independent pathways [8]. The diffusion pathway is considered relatively minor and cannot be up regulated [42]. However, non-specific diffusion may cause cell lysis and toxicity.

CS-6253 stimulated cholesterol efflux via ABCA1 in a concentration dependent manner in J774 cells (**S1 Fig**). The *K*_*m*_ value for cholesterol efflux by CS-6253 approached that of apo A-I, indicating that the single helix CS-6253 peptide stimulated cholesterol efflux via ABCA1 in J774 cells efficiently. In murine macrophages, extracellular accumulation of endogenously synthesized apo E promotes ABCA1 cholesterol efflux [43, 44] in addition to that derived by CS-6253-mediated cholesterol efflux *in-vitro*. To overcome this experimental limitation, our experiments were also conducted in BHK cells expressing ABCA1 as they do not secrete apo E [45]. We found that CS-6253 promotes cholesterol efflux with a *K*_*m*_ molar efficiency approximating that of apo E consistent with previous results using peptides fragments from the apo E carboxyl terminus domains (**Fig 2B**) [43]. The efficiency of CS-6253 in promoting ABCA1-mediated cholesterol and phospholipid efflux from murine J774 cells is in keeping with other apolipoprotein peptides [7, 29] (**S2 Fig**). The enhanced cholesterol and phospholipid efflux by CS-6252 is likely related to the increased amphipathicity of CS-6253 helix, thereby increasing its lipid affinity. Many apolipoproteins stimulate ABCA1 lipid efflux, suggesting common structural features may govern the efflux process [46]. Class A amphipathic α-helices are involved in lipid binding [7, 27, 46–48]. The efficiency of stimulated cholesterol and phospholipid efflux by mimetic peptides correlates with the lipid affinity [49]. These results suggest that apo mimetic peptides, as well as apo A-I first interact with the cell to form protein/phospholipid complexes that can accept cholesterol and form nHDL particles [50]. Moreover, CS-6253 is as effective as apo A-I in promoting cholesterol efflux from THP-1 macrophages derived foam cells expressing ABCA1 (**Fig 2C**). Cholesterol efflux caused net decrease but not clearance of stored CE in THP-1 cells (**Fig 2E**). Consequently, stimulation of CE hydrolysis by CS-6253 does not appear to be as quantitatively important in human cell THP-1 macrophages as apo A-I [51]. Only a proportion of FC was mobilized away from the CE cycle after apoA-I or CS-6253-cell interactions (29% and 11%, respectively; P<0.05). A similar ability was demonstrated with D-4F at same molar ratio for 24h [52]. This finding may be relevant *in-vivo* as aortic lesions are decreased with apoA-I infusions [53, 54]. We confirmed also that ABCA1 is expressed in THP-1 macrophages-derived foam cells (**Fig 2D**) [55].

Molecular interaction between CS-6253 and ABCA1 was investigated by chemical crosslinking. ABCA1 naturally dimerize with a possible higher order of oligomerization on the PM (**S3 Fig, left**) **[15].** CS-6253 did not impair oligomerization of ABCA1 (**S3 Fig**). This may be important as ABCA1 oligomerization is thought to serve as scaffold for apo A-I molecules to bind, interact and facilitate lipid efflux to apo A-I, thus allowing the formation of nascent LpA-I (nHDL) particles [15, 56–58].

To gain further insight into CS-6253 interaction with ABCA1, we performed competition assays. Our result suggests a direct interaction between CS-6253 and ABCA1, as observed for native apo A-I. However, ABCA1 interaction is not specific for apo A-I [59], but can occur with apolipoproteins that contain amphipathic helical domains such as apo E [16, 57] and also CS-6253. The present data indicates that the peptide is as efficient a competitor for the binding of apoA-I to ABCA1 as full length apoE. Moreover, CS-6253 competes five times more effectively than ATI-5261 with apo A-I for binding to ABCA1 (**Fig 3**). The amphipathic helical motif of CS-6253 may stabilize ABCA1 against proteolytic degradation in keeping with previous data from other mimetic peptides [60].

The peptide induced desorption of cholesterol and phospholipids from lipid rafts and non-rafts for nHDL biogenesis (**S4 Fig**). Differences observed for the desorption of ^3^[H]cholesterol and ^3^[H]choline phospholipids onto apo A-I or CS-6253 at 45 min, 6 hours and 12 hours support the concept that the plasma membrane plays a pivotal role in phospholipid and cholesterol desorption at the PM micro-domain level [58, 61]. This mechanism is in line with the concept of micro-solubilization model of membrane micro-domains proposed by Vedhachalam *et al*. [62]. Data from the apo A-I mimetic peptide 4F revealed lipid rafts disruption in the process of cholesterol and phospholipid efflux [63]. Here we report that CS-6253 exhibit non selective desorption of PC species and more efficient desorption than apo A-I. In fact, apo A-I appears to favor more non-raft desorption (**Fig 4A and 4B** *inset*) as previously reported [35]. The PC removal by CS-6253 appears independent of translocase activity of ABCA1. The SM species are removed from both raft and non-raft micro domains on to CS-6253 analogous with apoA-I (**Fig 4C and 4D** *inset*). This CS-6253 effect may be important as it has been suggested that similar modulation of lipid rafts by apo A-I or 4F peptide potentially inhibit pro-inflammatory gene expression, suggesting anti-inflammatory role of these mimetic peptides [63, 64]. Resemblance between natural physiology and CS-6253 was also supported by the finding that CS-6253 promoted the assembly of HDL particles with diameters similar to that of nascent apoA-I particles [65, 66].

ABCA1 binding and micro-solubilization of PM lipids, are key steps in ABCA1-dependent cholesterol efflux to CS-6253. However, the nature of the interaction between apolipoprotein peptides and ABCA1 is still unclear [5]. We speculate that a binding domain structure on CS-6253 interacts with ABCA1, and its amphipathic α-helix, leading to the micro-solubilization of PM, in turn facilitating the formation of lipid-rich HDL particles.

The nHDL CS-6253 particles generated by ABCA1 cells were not efficient acceptors for cholesterol when incubated with ABCG1 in contrast to nHDL-apo A-I. These studies also showed that CS-6253 may generate efficiently lipidated HDL particles through ABCA1 that are not necessarily subsequently lipidated by ABCG1. ABCG1 transporter, unlike ABCA1, prefers phospholipids-rich acceptor particles [67]. Decreased efficiency of ABCG1 efflux could be also attributed to the small ABCA1 mediated generation of intermediate-sized α-3 HDL-CS-6253 (~ 9 nm) (**Fig 5A and 5B)**, as this fraction is proposed to be the most efficient one in interacting with the ABCG1 to promote cellular cholesterol efflux [68].

To further characterize the efflux capacity of CS-6253 in human plasma; nHDL-CS-6253, nHDL-apoA-I and apoA-I were used under similar experimental conditions (**Table 1**). We found that lipid free CS-6253 is a preferred substrate to ABCA1 compared with nHDL-CS-6253 in plasma, in accordance with what was reported for lipid poor apoA-I [69, 70]. Plasma LCAT and PLTP are required for HDL remodeling [7, 8, 20, 32]. On a molar basis, CS-6253 was more efficient than the previously described ATI-5261 analogue (12) and as efficient as native apo A-I in activating LCAT *in-vitro*, in forming mature HDL in plasma (**S10 Fig**). It is well documented that LCAT plays a pivotal role in the RCT pathway by maintaining a concentration gradient of free cholesterol between cells membranes and HDL [71]. Sequence homology between CS-6253 and apoA-I may be a contributing factor for the increase in LCAT activation [72]. Previous studies of 37pA and 18A apolipoprotein mimetics revealed greater LCAT activity when compared with apoA-I [73, 74]. However, data from D-4F peptide (18 aa) showed no LCAT activity *in-vitro*, but D-4F activated LCAT in mice [75]. Other factors, such as SM content of HDL may modulate LCAT activity [76, 77]. The ability of CS-6253 to desorb SM species from PM may also contribute to its LCAT activation properties (**Fig 4D,** *inset*). Further lipidomic studies on CS-6253 HDL particles will be required to address this issue.

**Fig 11:**
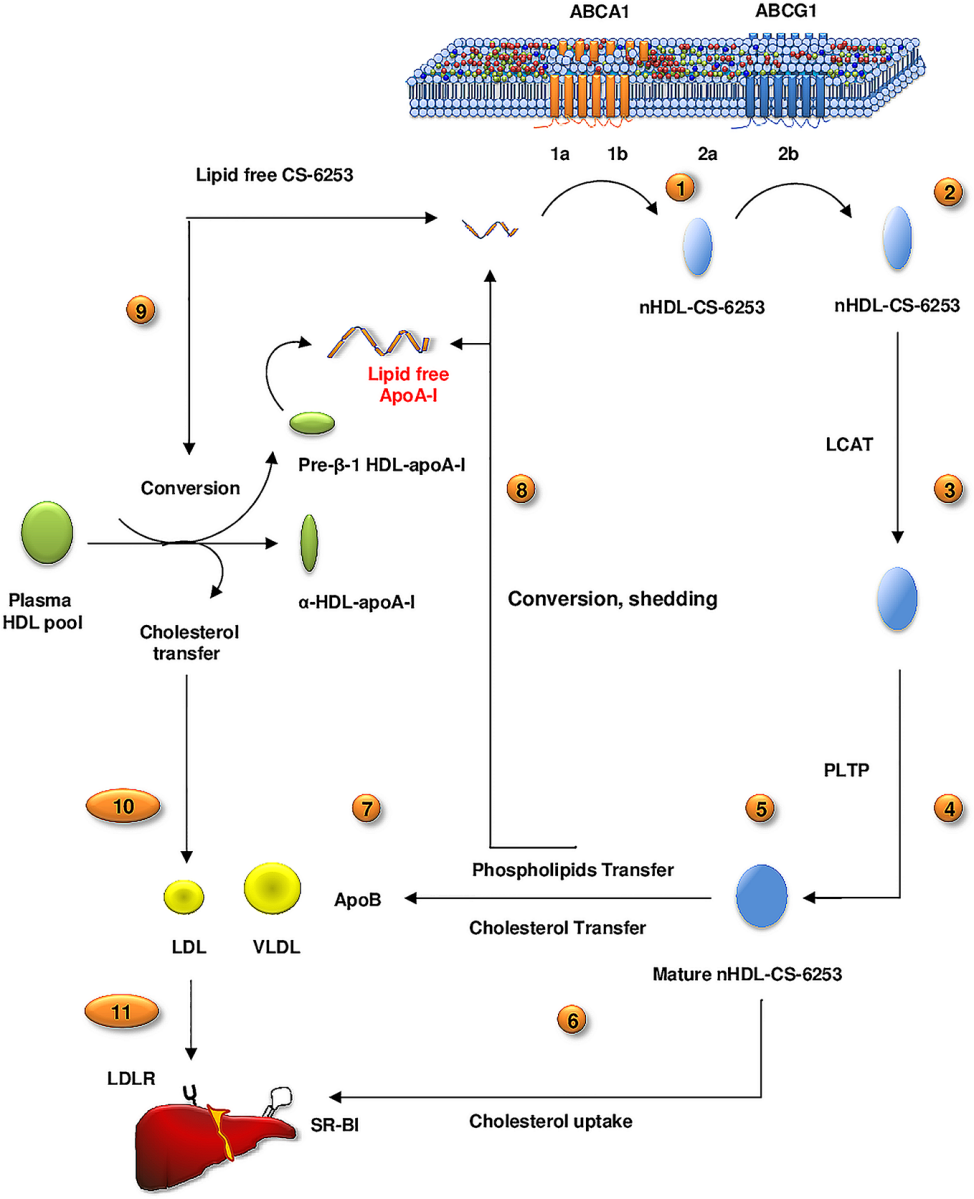
A proposed *in-vitro* model for lipid free CS-6253 lipidation and remodelling in RCT pathway. 1). ABCA1 interaction: a. ABCA1 oligomerization, b. Binding to ABCA-1. 2). Lipid efflux process: a. PM microdomains lipid desorption, b. ABCA1 and ABCG1 induction of lipid efflux and nHDL-CS-6253 formation, 3–4) HDL-CS-6253 remodelling by LCAT/PLTP in plasma, 5) generation of mature HDL-CS-6253, 6) Cholesterol uptake by liver via SR-BI, and 7) lipid transfer to both apoB-containing particles and the plasma HDL resident pool. 8) Although the mechanisms underlying this process is presently ambiguous, it is possible that nHDL-CS-6253 remodelling may lead to loss or ‘shedding’ of CS-6253 from HDL-CS-6253 in plasma as they are actively delipidated of phospholipids by PLTP to yield lipid poor apo A-I precursor of RCT activation. 9) Alternatively lipid free CS-6253 associates with αHDL-apo A-I species leading to preβ1-apo A-I formation, that can also close the loop and initiate again RCT. 10) in this process, the peptide transfer cholesterol to apoB particles, and 11) cholesterol is delivered to liver by the LDLR.

When ^3^[H]choline nHDL-CS-6253 is incubated in normolipidemic plasma with the PLTP stimulator AEBSF, the transfer of phospholipids is accelerated to apo B lipoproteins (**S11 Fig**). CS-6253 affects HDL remodeling physiologically in plasma. However, this observation requires confirmation *in-vivo*. Active remodeling of CS-6253 in plasma offers a mechanistic explanation for the elevation of ABCA1 dependent cholesterol efflux. As illustrated initially in (Fig **7D**), CS-6253 modulated preβ1-HDL generation in plasma. Similar data was reported for 4F *in-vitro* showing that increase in preβ-1 HDL formation is observed after a little as 1 minute in human plasma [78]. However, whether it does this *in vivo* with high affinity is unclear [5]. In our experiments, we found almost complete conversion of α to preβ-1 HDL by CS-6253 that was achieved after 5min (**Fig 8A and 8C**). Furthermore, analysis of the Lineweaver-Burk double reciprocal plot (**Fig 7C**, *inset*) reveals that CS-6253 decreased the app*Km* for cellular cholesterol efflux, when compared to plasma apo A-I alone. This finding is consistent with preβ-1 being responsible for the elevated capacity of plasma to stimulate cholesterol efflux via ABCA1 [9, 40, 41, 79], and suggests that similar effects might be observed by CS-6253 infusion *in vivo*. We propose that CS-6253 induction of preβ1-HDL formation and efficient efflux are mediated through a mechanism that includes binding of the peptide to HDL particles that displace apoA-I from α- HDL species to form preβ1-HDL in plasma. At the same time this mechanism is associated with dynamic transfer of cholesterol and phospholipids to apoB lipoproteins (**Fig 6B)**. This data is in keeping with cholesterol being transferred to LDL species as shown by FPLC profiles (**Fig 6C**), showing that the peptide in the apoB lipoproteins associate more with LDL more than with VLDL species (**Fig 6D**). Interestingly, CS-6253 associates with LDL and VLDL lipoproteins in plasma *in-vitro*. The significance of this finding remains unclear, but could reflect enhanced transfer of esterified cholesterol onto apoB-containing lipoproteins, and thus favoring HDL remodeling and RCT. This is in contrast to peptide 5A *in-vivo*, showing that 5A promotes transfer of cholesterol from LDL to HDL [9]. It is possible that in a closed *in-vitro* system the generation of preβ1-HDL decrease the storage capacity of HDL with transfer of HDL-C to other lipoprotein particles. Further studies are thus required *in-vivo* to better define the lipid transfer process from nHDL-CS-6253 to apo B particles, and clearance via the LDL receptor. Here, we show that preβ1-HDL particles are generated *de novo* by interaction between CS-6253 and α-HDL particles in plasma. These results, suggest that the peptide could enhance endogenous nHDL formation, thereby expanding the pool of lipid-poor apoA-I to exert potential anti atherosclerotic effects. Transient elevation of lipid poor apo A-I is thought to be clinically beneficial in removing cholesterol from the artery wall [80]. Interestingly, exogenous lipid-free ^125^I-apoA-I appears in pre-existing preß-migrating HDL species in normolipidemic plasma (**Fig 9B and 9C**). This association was conserved over the 4 h time course of the study. Within 5 min, lipid-free apoA-I incorporate rapidly in α–migrating HDLs in consistence with previous studies *in-vitro* and *in vivo* [20, 81]. The rapidity of this process may provide an explanation for the difficulty to document an increase in preβ-migrating apoA-I observed with the peptide in normal plasma. Additionally preβ-2 HDL particles seem unaffected by CS-6253 in plasma (**Figs 7D and 8A, 8B, 8C and 8D**). However, the detailed function of these particles has not been recognized yet [82]. Mechanism(s) of actions of apolipoprotein mimetic peptides in plasma remain unclear [5], and a detailed model is proposed in **Fig 11**illustrating CS-6253 effects in plasma. The uptake of cholesterol by the liver represents the final step in the RCT pathway. We have shown that apoA-I and CS-6253 promoted total cholesterol uptake in SR-BI expressing Fu5AH hepatoma cells (**Fig 10B**).

We provide evidence that CS-6253 enhances key steps of RCT *in-vitro*, in agreement with previous data on apolipoprotein peptides D-4F and 5A [9, 83]. Also, we present evidence demonstrating molecular interactions between ABCA1 and CS-6253 in a manner similarly to apo A-I [25, 35, 57, 84]. In addition, lipid free CS-6253 potentially modulates HDL metabolism more than nHDL-CS-6253 in plasma *ex-vivo*, suggesting that lipid free CS-6253 may have a therapeutic potential in man. Our data for CS-6253 peptide properties indicate feasibility of CS-6253 as a potential therapeutic agent—much more so than for the previously reported ATI-5261 peptide [27]. We previously showed that ATI-5261 possess efficient RCT properties *in-vitro* [11], with less efficiency in binding to ABCA1 than the CS-6253 analogue. The nHDL-CS-6253 like particles in human plasma are more efficient than those generated by ATI-5261 in activating LCAT and delivering cholesterol to hepatic cells through SR-BI. Thus, CS-6253 generates more efficient nHDL particles able to drive efficiently RCT process *in-vitro*. Indeed, ATI-5261 has unwanted *in vivo* toxicity at elevated doses limiting the therapeutic utility. The comparison between CS-6253 and ATI-5261 clearly demonstrate that ABCA1 cholesterol efflux properties are separate and apart from muscle toxicity. The findings support the feasibility of ABCA1 as a safe and efficient target for the treatment of atherosclerosis conditions.

In summary, as illustrated in (**Fig 11**), we have shown that CS-6253 promotes key steps in the RCT pathway. CS-6253 binds to ABCA1 and competes efficiently with apo A-I, conserves the oligomeric structure of ABCA1, and generates nascent HDL-like particles. In plasma, these CS-6253 HDL particles undergo remodeling by LCAT and PLTP and serve as efficient donors of cholesterol to hepatocytes via the SR-BI receptor. Our data also suggests that CS-6253 added directly to human plasma promotes ABCA1-dependent cellular cholesterol efflux efficiently. When incubated with human plasma CS-6253 binds to HDL and LDL and increased the transfer of cholesterol from HDL to apoB particles, predominantly to LDL species. The formation of preβ-1 HDL with the cycling of apo A-I between the preβ and α-HDL are proposed here to be crucial mechanisms of RCT facilitated by ABCA1 interaction. This mechanism may play an important role in the protection against atherosclerosis by the CS-6253 ABCA1 agonist peptide.

## Limitations

A key limitation of this study is that it was conducted in *in-vitro* system of RCT that may not allow a full understanding of the complex lipoprotein metabolism and physiology in man. More animal studies are essential to explore the anti-atherogenic properties of this peptide. Furthermore, we did not explore the pleiotropic effects of HDL on inflammatory, oxidation or endothelial cell biomarkers. Additional work will be required to target HDL functions and its regulations [33], that might provide valuable guidance for clinical trials and applications.

## Supporting Information

S1 Appendix Supplementary appendix. (DOCX)

S1 Fig In-vitro cholesterol efflux activity of CS-6253. Dose dependent ABCA1 mediated cholesterol efflux in cAMP treated J774 cells. Kinetic parameters for ABCA1-mediated cholesterol efflux from J774 cells to apo A-I or CS-6253 peptide are as follows: Apo A-I: *K_m_* = 3.82±1.81 μg/ml (0.15±0.05 μM), *V_max_* = 6.90±1.06% efflux/6h, and relative catalytic efficiency: *V_max_/K_m_* = 1.80. CS-6253: *K_m_* = 0.17±0.10 μg/ml (0.54±0.10 μM), *V_max_* = 6.42±0.17% efflux/6h, and relative catalytic efficiency: *V_max_/K_m_* = 37.76.
(TIF)

S2 Fig Phospholipid efflux from BHK cells expressing ABCA1 to increasing concentrations of lipid free CS-6253 and apo A-I. Baby hamster kidney (BHK) cells were labeled with ^3^[H]-choline. ABCA1 expression was induced by treating BHK cells with 10 nM mifepristone. Cells were then changed into fresh DMEM medium [no apolipoprotein A-I (apoA-I)] and incubated with apoA-I or CS-6253 (0.96 uM). Medium was collected after 2 h. Medium and cell-associated 3H radioactivity was counted and presented as percentage of cholesterol in the medium relative to the total cholesterol (medium and cell-associated).Kinetic parameters for ABCA1-mediated phospholipids efflux from BHK-ABCA1 cells to apo A-I: *K_m_* = 3.65±0.49 μg/ml (0.14±0.02 μM), *V_max_* = 0.77±0.02% efflux/2h, and relative catalytic efficiency: *V_max_/K_m_* = 0.21. CS-6253: *K_m_* = 1.03±0.15 μg/ml (0.33±0.05 μM), *V_max_* = 0.96±0.02% efflux/2h, and relative catalytic efficiency: *V_max_/K_m_* = 0.93. Results represent the mean of triplicates ± SD, n = 3.
(TIF)

S3 Fig Chemical cross-linking of ABCA1 in intact BHK cells expressing ABCA1. BHK cells in 100-mm diameter dishes were stimulated with 10 nM mifepristone for 18–20 h. BHK cells expressing ABCA1 (100 μg of total protein) in the presence of apo A-I or CS-6253 at same molarity (0.96 μM) were cross-linked or not with 500 μM DSP. Cells were then lysed at 4°C with lysis buffer containing 0.5% *n*-dodecylmaltoside in the presence of a protease inhibitor mixture followed by low speed centrifugation to remove cell debris. The supernatants were treated or not with 50 mM DTT for 30 min at 37°C and then separated by SDS-PAGE (4–22.5%) in duplicate. After electrophoresis, ABCA1 was detected by an anti-ABCA1 antibody. GAPDH was used as loading control.
(TIF)

S4 Fig Apo A-I desorbs cholesterol from raft and nonraft domains. BHK-ABCA1 cells were labeled with ^3^[H]cholesterol or ^3^[H]choline for 48 h, followed by stimulation with mifepristone for 18–20h as described in experimental procedures in the online-only Supplementary appendix, S1 Appendix. Cells were then incubated for 45 min with apo A-I or CS-6253.
(TIF)

S5 Fig CS-6253 desorbs cholesterol from raft and nonraft domains. BHK-ABCA1 cells were labeled with ^3^[H]cholesterol or ^3^[H]choline for 48 h, followed by stimulation with mifepristone for 18–20h as described in experimental procedures in the online-only Supplementary appendix, S1 Appendix. Cells were then incubated for 45 min with apo A-I or CS-6253.
(TIF)

S6 Fig Apo A-I desorbs phospholipids from raft and nonraft domains. BHK-ABCA1 cells were labeled with ^3^[H]cholesterol or ^3^[H]choline for 48 h, followed by stimulation with mifepristone for 18–20h as described in experimental procedures in the online-only Supplementary appendix, S1 Appendix. Cells were then incubated for 45 min with apo A-I or CS-6253.
(TIF)

S7 Fig CS-6253 desorbs phospholipids from raft and nonraft domains. BHK-ABCA1 cells were labeled with ^3^[H]cholesterol or ^3^[H]choline for 48 h, followed by stimulation with mifepristone for 18–20h as described in experimental procedures in the online-only Supplementary appendix, S1 Appendix. Cells were then incubated for 45 min with apo A-I or CS-6253. After lipid extraction, ^3^[H]cholesterol in each fraction was assessed for radioactivity. Radioactivity appearing in fractions corresponding to raft (1–5) and nonraft (6–10) material was pooled, and desorption of ^3^[H]cholesterol and ^3^[H]choline, from raft versus nonraft in the presence of apo A-I or CS-6253 was expressed as a percentage of control (100%, in the absence of acceptor) (**S4 to S7** *inset*). Results shown are representative of three independent experiments. *P < 0.05 by Student's t-test.
(TIF)

S8 Fig *In-vitro* time-dependent ABCG1-mediated cholesterol efflux activity to nHDL-CS-6253 particles generated by BHK cells expressing ABCA1. Unlabeled BHK cells expressing ABCA1 were treated with 0.96 μM lipid free apo A-I or CS-6253 for 18 h as described in experimental procedures in the online-only Supplementary appendix, S1 Appendix. Then the medium was collected and added to 3[H]cholesterol-labeled ABCG1 expressing cells in a time-dependent fashion (0–6h), and assayed for cholesterol efflux (only 6h time point is shown).
(TIF)

S9 Fig *In-vitro* ABCG1-mediated cholesterol efflux activity to lipid free CS-6253 and nHDL-CS-6253 particles generated by BHK cells expressing ABCA1. Unlabeled ABCA1 expressing cells were treated with increasing concentrations of apo A-I or CS-6253 for 18 h. The medium from unlabeled ABCA1 cells treated as described in experimental procedures, centrifuged to remove cellular debris, added to ^3^[H]cholesterol-BHK ABCG1 expressing cells for 6 h, and assayed for efflux as described in the online-only Supplementary appendix, S1 Appendix. Lipid free peptide and apo A-I are also tested against ABCG1 expressing cells for 6h cholesterol efflux. (We selected 1 μM nHDL mimetic or nHDL-apo A-I or lipid free peptide and apo A-I for the figure). Kinetic parameters for ABCG1-mediated cholesterol efflux to nHDL-apo A-I or CS-6253 are as follows: nHDL-Apo A-I: *K_m_* = 2.63±0.38 μg/ml (0.10±0.01 μM), *V_max_* = 16.23±0.61% efflux/6h, and relative catalytic efficiency: *V_max_/K_m_* = 6.17. nHDL-CS-6253: *K_m_* = 2.59±0.65 μg/ml (0.76±0.18 μM), *V_max_* = 4.61±0.65% efflux/4h, and relative catalytic efficiency: *V_max_/K_m_* = 1.77. Kinetic parameters for ABCG1-mediated cholesterol efflux to lipid free peptide and apo A-I are as follows: Apo A-I: *K_m_* = 1.7±0.50 μg/ml (0.06 ± 0.01 μM), *V_max_* = 0.96 ± 0.01% efflux/6h, and relative catalytic efficiency: *V_max_/K_m_* = 0.56. CS-6253: *K_m_* = 8.33±4.36 μg/ml (0.18±0.15 μM), *V_max_* = 0.99±0.19% efflux/6h, and relative catalytic efficiency: *V_max_/K_m_* = 0.11. Efflux of ^3^[H]cholesterol is shown as means ± SD of triplicate experiments.
(TIF)

S10 Fig Dynamics of esterification of nascent LpCS-6253. nHDL-CS-6253 particles were labelled with cell derived ^3^[H]cholesterol and incubated with a normolipidemic plasma at a ratio of (1μg peptide: 10μg plasma apo A-I). LCAT activity was determined after incubation for 1h at 37°C in the presence or absence of 2 mM DTNB. After lipid extraction, ^3^[H]cholesterol (unesterified) and ^3^[H]CE from plasma were separated by TLC and assayed for radioactivity as described in the online-only Supplementary appendix, S1 Appendix. LCAT activity was measured as CE divided by cholesterol/h. Results are mean (±SD) of triplicate experiments. *P<0.05.
(TIF)

S11 Fig Dynamics of transfer of nascent LpCS-6253. Radiolabelled ^3^[H]-choline nascent–like HDL lipoprotein apo A-I, CS-6253 or were incubated with normolipidemic plasma for 6 h at 37°C in the presence or the absence of AEBSF as indicated in methods in the online-only Supplementary appendix, S1 Appendix. After incubation, apoB was precipitated by PEG. ApoB containing particles fractions were dialyzed and PLTP transfer is calculated between nHDL and apoB fractions. Control (open bar) consists of radiolabelled particles alone in PBS+BSA. Results are mean (±SD) of triplicate experiments. *P<0.05.
(TIF)
