# Supplementary material for: Novel Apo E-Derived ABCA1 Agonist Peptide (CS-6253) Promotes Reverse Cholesterol Transport and Induces Formation of preβ-1 HDL In Vitro
Source: PLoS One. 2015 Jul 24;10(7):e0131997. doi: 10.1371/journal.pone.0131997 (PMC4514675; doi:10.1371/journal.pone.0131997)
Supplement: S1 Appendix — (DOCX) [file pone.0131997.s001.docx]

**SUPPLEMENTARY APPENDIX**

**Novel apo E-derived ABCA1 agonist peptide (CS-6253) Promotes Reverse Cholesterol Transport and induces formation of**

**preβ-1 HDL *in vitro***

Anouar Hafiane^1^, John K. Bielicki^2^, Jan O. Johansson^3^ and Jacques Genest^1^

^1^Cardiovascular Research Laboratories Laboratory, Research Institute of the McGill University Health Centre, Montréal, Québec H4A 3J1, Canada

^2^Lawrence Berkeley National Laboratory, Donner Laboratory, MS1-267, Berkeley, CA

^3^Artery Therapeutics, San Ramon CA

**TABLE OF CONTENTS**

Page 3 *Supplementary Methods*

Cellular cholesterol efflux capacity assays

Page 4 *Supplementary Methods*

Cellular phospholipid efflux assays

Page 5 *Supplementary Methods*

Cross-linking experiments

Page 6 *Supplementary Methods*

Ultracentrifugation sucrose gradient fractionation protocol

Page 7 *Supplementary Methods*

Lecithin: Cholesterol Acyl Transferase (LCAT) activity assay

Page 8 *Supplementary Methods*

Phospholipid Transfer Protein (PLTP) activity assay

***Supplementary Methods***

**Cellular cholesterol efflux capacity assays**

All cell culture incubations were performed at 37ºC in a humidified 5% CO_2_. Cells (J774, or BHK overexpressing human ABCG1) were labeled with 2 μCi/ml ^3^H-free cholesterol (Perkin Elmer, Norwalk, Connecticut) for 24 h. Simultaneously J774 cells were incubated with 2 μg/ml ACAT inhibitor (Sandoz, Sigma, Oakville, Canada). Afterward, J774 and BHK-ABCG1 were stimulated with 0.5 mmol/l (cpt-cAMP) (2) or 10 nmol/l mifepristone for 18-20 h, respectively (1). Cells were incubated with various doses of acceptor in media for 6 h for J774 macrophages and BHK-ABCG1 cells. Media were collected, centrifuged at 3.000 *g* for 5min to remove cell debris, and counted for radioactivity. Cells were lysed in 0.5 ml of (0.1N) NaOH overnight with constant shaking. Further, 0.2 ml cells lysates were mixed with scintillation liquid and counted for radioactivity. Efflux from stimulated cells without acceptor was subtracted from total efflux to determine net efflux. Cholesterol efflux was calculated according to the following formula as the percentage of ^3^H-cpm medium/(^3^H-cpm medium + ^3^H-cpm cells) x 100%. Efflux efficiency (*K_m_*) was calculated using the Michaelis-Menten equation (Graph-Pad Prism6 software) respectively (**S1, S8 and S9 Figs.)**

*References:*

1. Oram, J. F., Vaughan, A. M., and Stocker, R. (2001) ATP-binding cassette transporter A1 mediates cellular secretion of alpha-tocopherol. The Journal of biological chemistry 276, 39898-39902
2. Hafiane, A., Jabor, B., Ruel, I., Ling, J., and Genest, J. (2014) High-density lipoprotein mediated cellular cholesterol efflux in acute coronary syndromes. Am J Cardiol 113, 249-255.
3. Doonan, R. J., Hafiane, A., Lai, C., Veinot, J. P., Genest, J., and Daskalopoulou, S. S. (2014) Cholesterol efflux capacity, carotid atherosclerosis, and cerebrovascular symptomatology. Arteriosclerosis, thrombosis, and vascular biology 34, 921-926

*Supplementary Methods*

**Cellular phospholipid efflux assays**

Cells were seeded (50×103 cells) in 24 well plates containing DMEM plus 10% FBS with 3 μCi/ml ^3^H-choline chloride (Perkin Elmer, Norwalk, Connecticut), for 72 hours as previously reported with minor changes [1,2]). Cells were then put into induction medium for 18–20 h (10 nM mifepristone). This was followed by incubation with increased molar doses of apo A-I and CS-6253 for 2 h at 37°C. Efflux medium was collected, and cellular lipids were extracted with hexane/isopropanol (3:2 v/v), while total phospholipids from the medium were extracted in chloroform /methanol (2:1 v/v). Efflux was determined as percent of total ^3^[H] Phospholipids in the medium. All results were conformed at least twice. (**S2 Fig.**)

*References:*

1. Nandi, S., Ma, L., Denis, M., Karwatsky, J., Li, Z., Jiang, X. C., and Zha, X. (2009) ABCA1-mediated cholesterol efflux generates microparticles in addition to HDL through processes governed by membrane rigidity. Journal of lipid research 50, 456-466.
2. Marcil, M., Bissonnette, R., Vincent, J., Krimbou, L., and Genest, J. (2003) Cellular phospholipid and cholesterol efflux in high-density lipoprotein deficiency. Circulation 107, 1366-1371

*Supplementary Methods*

**Cross-linking experiments**

Chemical cross-linking was performed as described previously ADDIN EN.CITE <EndNote><Cite><Author>Wang</Author><Year>2001</Year><RecNum>16</RecNum><DisplayText>(1)</DisplayText><record><rec-number>16</rec-number><foreign-keys><key app="EN" db-id="rsz92txte02xvgewsx9v9rpptsf52zzexeza">16</key></foreign-keys><ref-type name="Journal Article">17</ref-type><contributors><authors><author>Wang, N.</author><author>Silver, D. L.</author><author>Thiele, C.</author><author>Tall, A. R.</author></authors></contributors><auth-address>Division of Molecular Medicine, Department of Medicine, Columbia University, New York, New York 10032, USA. nw30@columbia.edu</auth-address><titles><title>ATP-binding cassette transporter A1 (ABCA1) functions as a cholesterol efflux regulatory protein</title><secondary-title>J Biol Chem</secondary-title><alt-title>The Journal of biological chemistry</alt-title></titles><periodical><full-title>J Biol Chem</full-title><abbr-1>The Journal of biological chemistry</abbr-1></periodical><alt-periodical><full-title>J Biol Chem</full-title><abbr-1>The Journal of biological chemistry</abbr-1></alt-periodical><pages>23742-7</pages><volume>276</volume><number>26</number><edition>2001/04/20</edition><keywords><keyword>ATP Binding Cassette Transporter 1</keyword><keyword>ATP-Binding Cassette Transporters/genetics/ physiology</keyword><keyword>Apolipoprotein A-I/ metabolism</keyword><keyword>Biological Transport/drug effects</keyword><keyword>Cell Line</keyword><keyword>Cholesterol/ metabolism</keyword><keyword>Cyclodextrins/pharmacology</keyword><keyword>Glyburide/pharmacology</keyword><keyword>Humans</keyword><keyword>Mutation</keyword><keyword>Phospholipids/metabolism</keyword></keywords><dates><year>2001</year><pub-dates><date>Jun 29</date></pub-dates></dates><isbn>0021-9258 (Print)&#13;0021-9258 (Linking)</isbn><accession-num>11309399</accession-num><urls/><electronic-resource-num>10.1074/jbc.M102348200</electronic-resource-num><remote-database-provider>NLM</remote-database-provider><language>eng</language></record></Cite></EndNote>(1) with minor modifications. Baby hamster kidney cells (BHK) cells expressing ABCA1 were grown to confluence in 100-mm diameter dishes and then stimulated with 10 nM mifepristone for 18-20 h in Dulbecco’s modified Eagle’s medium/ bovine serum albumin (DMEM/BSA) 0.2%. Cells were incubated with 0.93 μM of apo A-I or CS-6253 for 1h at 37ºC and then placed on ice for 15 min and washed three times with PBS. The dithiobis (succinimidylpropinate) (DSP) cross linker agent (Pierce, Rockford, IL), was dissolved immediately before use in Me_2_SO and diluted to 500 μM in PBS. Eight ml of DSP solution was added in each dish. Cells were then incubated at room temperature for 30 min; the medium was discarded, and the cells were washed twice with PBS. Cells were lysed at 4ºC with (20 mM Tris, 5 mM EDTA, and 5 mM EGTA; pH 7.5) containing 0.5% *n*-dodecylmaltoside, and the suspension was allowed to stand for 20 min on ice for further lysis in the presence of a protease inhibitor mixture (Roche Diagnostic). After solubilization of cell proteins and centrifugation at 11,000 g, 4°C, for 10 min, the supernatants were treated or not with 50 mM dithiothreitol (DTT) for 30 min at 37°C, and then the samples were separated by SDS-PAGE (4–22.5%) as described previously ADDIN EN.CITE <EndNote><Cite><DisplayText>(2)</DisplayText><record></record></Cite></EndNote>(2). Protein concentration was determined by standard assay (Bio-Rad). ABCA1 was detected by an affinity-purified human anti-ABCA1 antibody (Novus Biologicals). The presence of ABCA1 oligomers was detected with chemiluminescence (ECL, Thermo Scientific, USA) by autoradiography by using XAR-2 Kodak film. (**S3 Fig.**).

*References:*

1. Wang, N., Silver, D. L., Thiele, C., and Tall, A. R. (2001) ATP-binding cassette transporter A1 (ABCA1) functions as a cholesterol efflux regulatory protein. *The Journal of biological chemistry* 276, 23742-23747
2. Denis, M., Haidar, B., Marcil, M., Bouvier, M., Krimbou, L., and Genest, J. (2004) Characterization of oligomeric human ATP binding cassette transporter A1. Potential implications for determining the structure of nascent high density lipoprotein particles. The Journal of biological chemistry 279, 41529-41536

*Supplementary Methods*

**Ultracentrifugation sucrose gradient fractionation protocol**

Sucrose density gradient fractionation was performed by ultracentrifugation (1,2). Cells were lysed at 4°C with TNE buffer (50 mM Tris-HCl (pH 7.5), 140 mM NaCl, 5 mM EDTA) containing 0.2% (v/v) Triton X-100 (Sigma-Aldrich, Oakville, Ontario, Canada), and protease inhibitor cocktail (Roche) for 30 min on ice followed by low-speed centrifugation (1.5 g for 10 min, 4ºC) to remove insoluble materials. Samples were mixed with an equal volume of 90% (w/v) sucrose in MBS (25 mM MES (pH 6.5), 150 mM NaCl) and overlaid with 35, 30, 25, and 5% (w/v) sucrose. The gradient was spun at 198,000 g in a Beckman SW41 rotor for 16 h. Ten fractions of 1ml were collected from top to bottom and analyzed for radioactivity, and lipid content ^3^[H]-FC and ^3^[H]-choline. (S**4, S5, S6, and S7 Figs.**).

*References:*

1. Li, X., and Donowitz, M. (2014) Fractionation of subcellular membrane vesicles of epithelial and non-epithelial cells by OptiPrep density gradient ultracentrifugation. Methods in molecular biology (Clifton, N.J.) 1174, 85-99.
2. Iatan, I., Bailey, D., Ruel, I., Hafiane, A., Campbell, S., Krimbou, L., and Genest, J. (2011) Membrane microdomains modulate oligomeric ABCA1 function: impact on apoAI-mediated lipid removal and phosphatidylcholine biosynthesis. Journal of lipid research 52, 2043-2055

*Supplementary Methods*

**Lecithin: Cholesterol Acyl Transferase (LCAT) activity assay**

The fractional cholesterol esterification rate (FER) of LCAT activity was assayed with standard methodology (1,2). The nHDL-apo A-I and nHDL-CS-6253 particles were prepared after radiolabelling of BHK-ABCA1 cells with ^3^[H]cholesterol (PerkinElmer Life Science) after 4 h incubation with lipid free apo A-I or CS-6253 (0.96 μM), media were collected under protease inhibitor and lipid-free apo A-I or CS-6253 were removed by ultrafiltration (spiral ultrafiltration cartridge, MWCO 50,000, Amicon). Cholesterol labeled nHDL particles, Lp-CS-6253 or LpA-I (1μg: 10 μg plasma apo A-I), are incubated in total plasma pooled from healthy control subjects for 1h at 37ºC (3). This ratio is selected based on the assumption that the in vivo nascent HDL pool represented ∼10% of total plasma apoA-I mass (4). LCAT activity in nHDL-apo A-I is used as positive control and inhibition of LCAT activity with (2 mM) 5,5′-Dithiobis(2-nitrobenzoic acid) (DTNB) from Sigma is used as a negative-control. Cellular lipids were extracted and ^3^[H]cholesterol and ^3^[H]cholesteryl esters (CE) were separated by TLC. The lipids ^3^[H]free cholesterol and ^3^[H]CE were located by exposure to iodine vapor, and were scraped into liquid scintillating vials and assayed for radioactivity. The values of LCAT activity are the mean (±SD) of triplicate measurement. The integrity and quality of isolated HDL-CS-6253 particles were verified by 2D-PAGGE analysis before and after incubation in total plasma, and used within 24 h. (**S10 Fig**).

*References:*

1. Dobiasova, M., and Frohlich, J. (1996) Measurement of fractional esterification rate of cholesterol in plasma depleted of apoprotein B containing lipoprotein: methods and normal values. *Physiol Res* 45, 65-73.
2. Frohlich, J., and Dobiasova, M. (2003) Fractional esterification rate of cholesterol and ratio of triglycerides to HDL-cholesterol are powerful predictors of positive findings on coronary angiography. *Clin Chem* 49, 1873-1880.
3. Bailey, D., Ruel, I., Hafiane, A., Cochrane, H., Iatan, I., Jauhiainen, M., Ehnholm, C., Krimbou, L., and Genest, J. (2010) Analysis of lipid transfer activity between model nascent HDL particles and plasma lipoproteins: implications for current concepts of nascent HDL maturation and genesis. *Journal of lipid research* 51, 785-797.
4. Batal, R., Tremblay, M., Krimbou, L., Mamer, O., Davignon, J., Genest, J., Jr., and Cohn, J. S. (1998) Familial HDL deficiency characterized by hypercatabolism of mature apoA-I but not proapoA-I. *Arteriosclerosis, thrombosis, and vascular biology* 18, 655-664

*Supplementary Methods*

**Phospholipid Transfer Protein (PLTP) activity assay**

Endogenous PLTP activity was determined as follows: Cell-derived ^3^[H]choline-labeled LpA-I and ^3^[H]choline-labeled LpCS-6253 were incubated with total plasma (10µg LpA-I: 100µg/ml of plasma apoA-I) versus (10µg LpCS-6253: 100µg/ml of plasma apoA-I) at 37°C for 6 h in the absence or presence of (10 mM) 4-(2-Aminoethyl)benzenesulfonyl fluoride hydrochloride from Sigma (AEBSF) (1,2). After incubation, plasma apo B was precipitated with an equal volume of 13% (PEG) 6000, and the lipids from the supernatants (HDL) and precipitates (apoB) were extracted (1). Transfer of ^3^[H]choline from nHDL-like particles to the apoB fraction was expressed in percentage of ^3^[H]choline transferred per hour per milliliter of plasma, and calculated as % pellet ^3^[H](cpm pellet/(cpm pellet + cpm supernatant)). The integrity and quality of isolated HDL-CS-6253 particles were verified by analysis with 2D-PAGGE before and after incubation in total plasma and used within 24 h. (**S11 Fig.**).

*References:*

1. Bailey, D., Ruel, I., Hafiane, A., Cochrane, H., Iatan, I., Jauhiainen, M., Ehnholm, C., Krimbou, L., and Genest, J. (2010) Analysis of lipid transfer activity between model nascent HDL particles and plasma lipoproteins: implications for current concepts of nascent HDL maturation and genesis. Journal of lipid research 51, 785-797.
2. Hafiane, A., Bielicki, J. K., Johansson, J. O., and Genest, J. (2014) Apolipoprotein E derived HDL Mimetic Peptide ATI-5261 Promotes Nascent HDL Formation and Reverse Cholesterol Transport in vitro. Biochim Biophys Acta. Oct;1842(10):1498-512.
