# Supplementary figures and images for: Novel Apo E-Derived ABCA1 Agonist Peptide (CS-6253) Promotes Reverse Cholesterol Transport and Induces Formation of preβ-1 HDL In Vitro
Source: PLoS One. 2015 Jul 24;10(7):e0131997. doi: 10.1371/journal.pone.0131997 (PMC4514675; doi:10.1371/journal.pone.0131997)

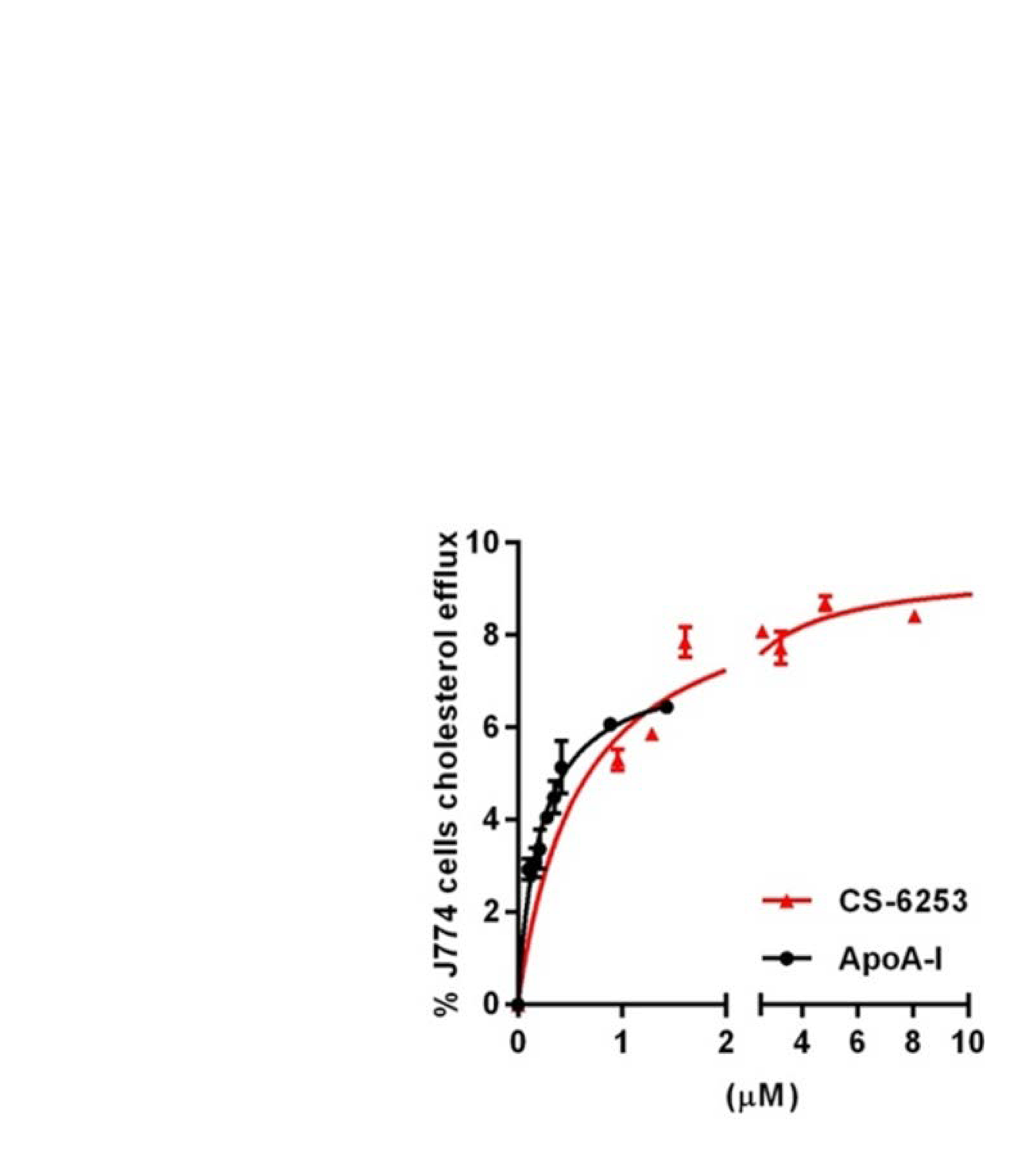

Supplement: S1 Fig — Dose dependent ABCA1 mediated cholesterol efflux in cAMP treated J774 cells. Kinetic parameters for ABCA1-mediated cholesterol efflux from J774 cells to apo A-I or CS-6253 peptide are as follows: Apo A-I: Km = 3.82±1.81 μg/ml (0.15±0.05 μM), Vmax = 6.90±1.06% efflux/6h, and relative catalytic efficiency: Vmax/Km = 1.80. CS-6253: Km = 0.17±0.10 μg/ml (0.54±0.10 μM), Vmax = 6.42±0.17% efflux/6h, and relative catalytic efficiency: Vmax/Km = 37.76. (TIF) [file pone.0131997.s002.tif]

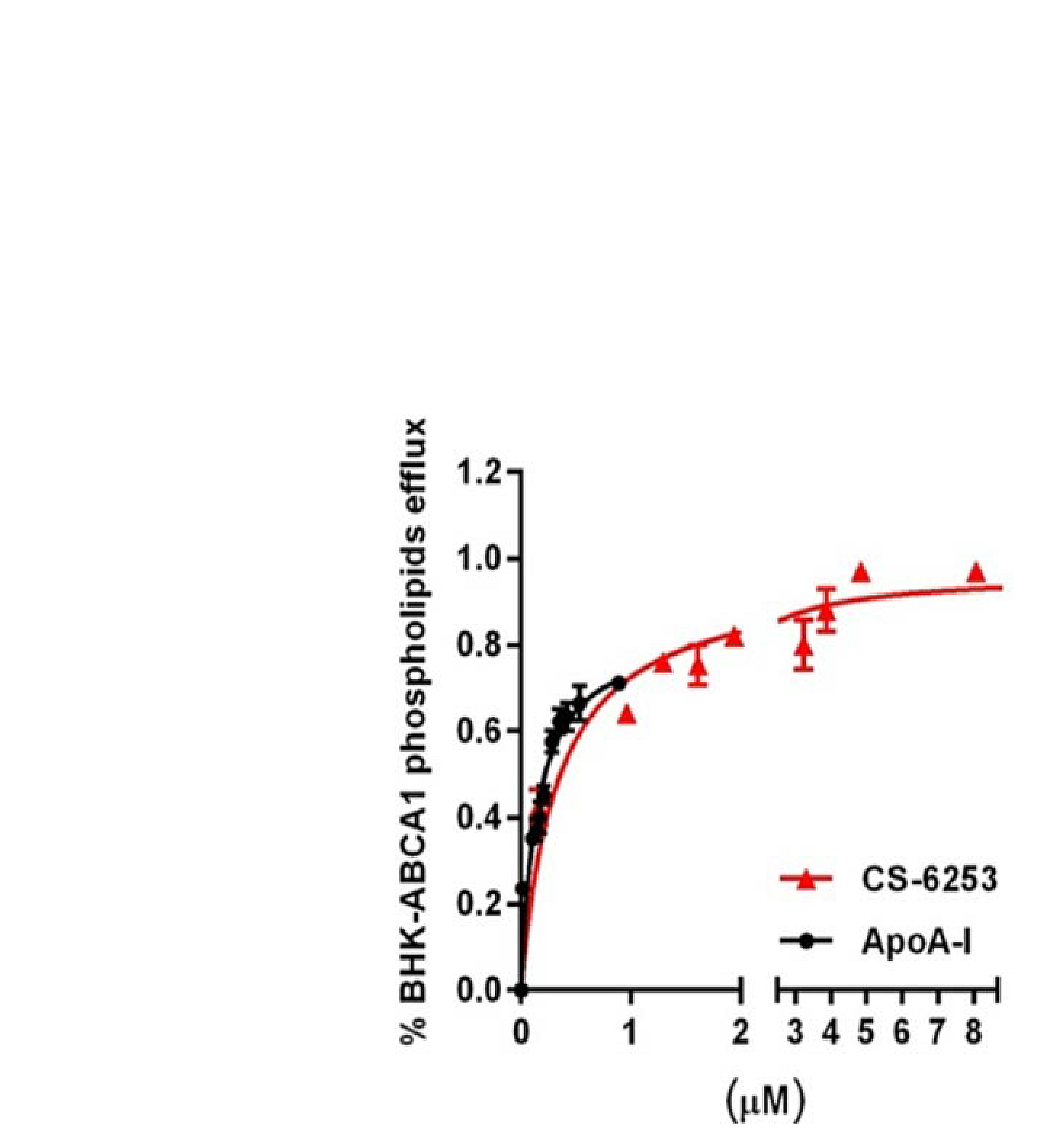

Supplement: S2 Fig — Baby hamster kidney (BHK) cells were labeled with 3[H]-choline. ABCA1 expression was induced by treating BHK cells with 10 nM mifepristone. Cells were then changed into fresh DMEM medium [no apolipoprotein A-I (apoA-I)] and incubated with apoA-I or CS-6253 (0.96 uM). Medium was collected after 2 h. Medium and cell-associated 3H radioactivity was counted and presented as percentage of cholesterol in the medium relative to the total cholesterol (medium and cell-associated).Kinetic parameters for ABCA1-mediated phospholipids efflux from BHK-ABCA1 cells to apo A-I: Km = 3.65±0.49 μg/ml (0.14±0.02 μM), Vmax = 0.77±0.02% efflux/2h, and relative catalytic efficiency: Vmax/Km = 0.21. CS-6253: Km = 1.03±0.15 μg/ml (0.33±0.05 μM), Vmax = 0.96±0.02% efflux/2h, and relative catalytic efficiency: Vmax/Km = 0.93. Results represent the mean of triplicates ± SD, n = 3. (TIF) [file pone.0131997.s003.tif]

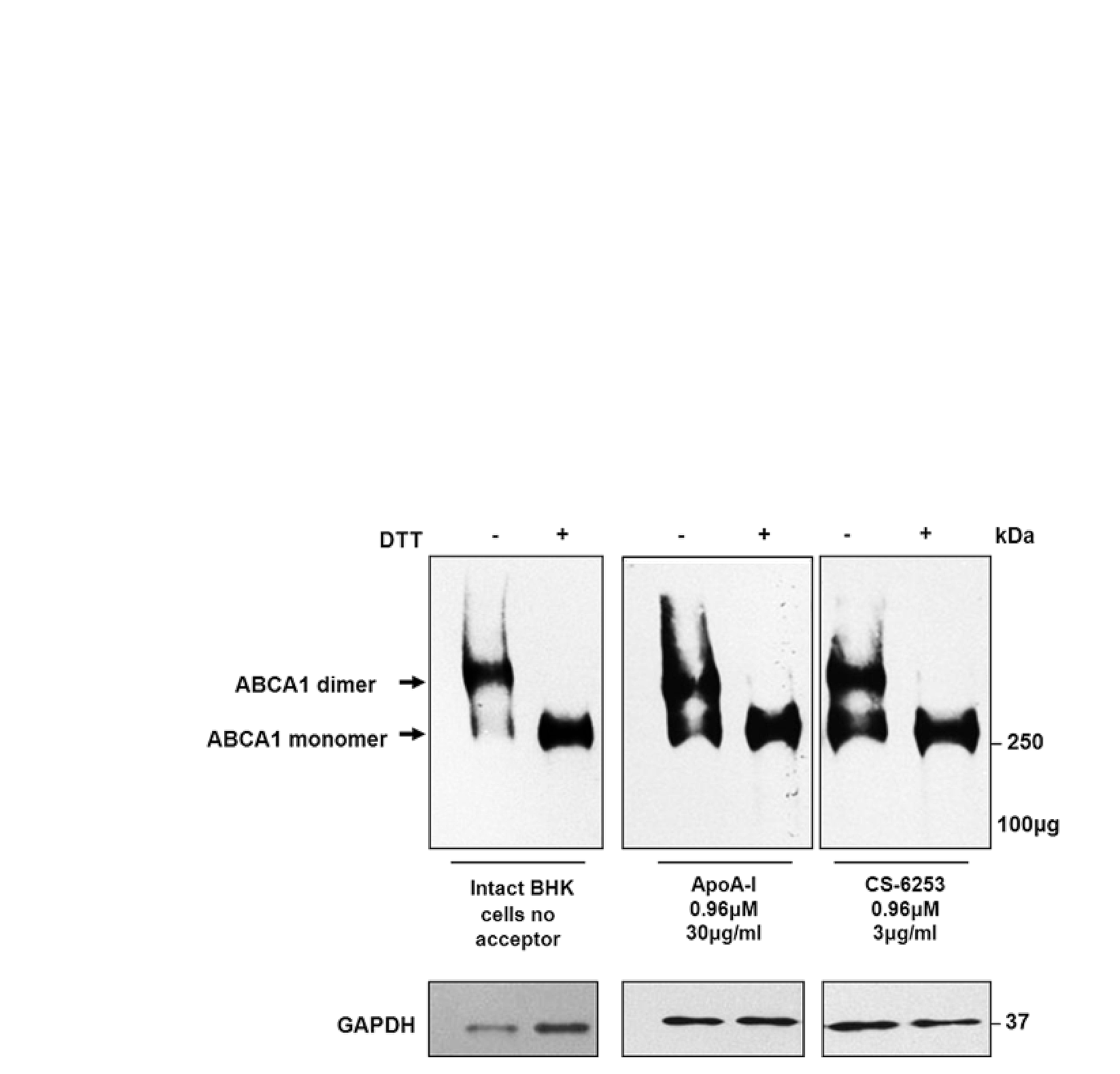

Supplement: S3 Fig — BHK cells in 100-mm diameter dishes were stimulated with 10 nM mifepristone for 18–20 h. BHK cells expressing ABCA1 (100 μg of total protein) in the presence of apo A-I or CS-6253 at same molarity (0.96 μM) were cross-linked or not with 500 μM DSP. Cells were then lysed at 4°C with lysis buffer containing 0.5% n-dodecylmaltoside in the presence of a protease inhibitor mixture followed by low speed centrifugation to remove cell debris. The supernatants were treated or not with 50 mM DTT for 30 min at 37°C and then separated by SDS-PAGE (4–22.5%) in duplicate. After electrophoresis, ABCA1 was detected by an anti-ABCA1 antibody. GAPDH was used as loading control. (TIF) [file pone.0131997.s004.tif]

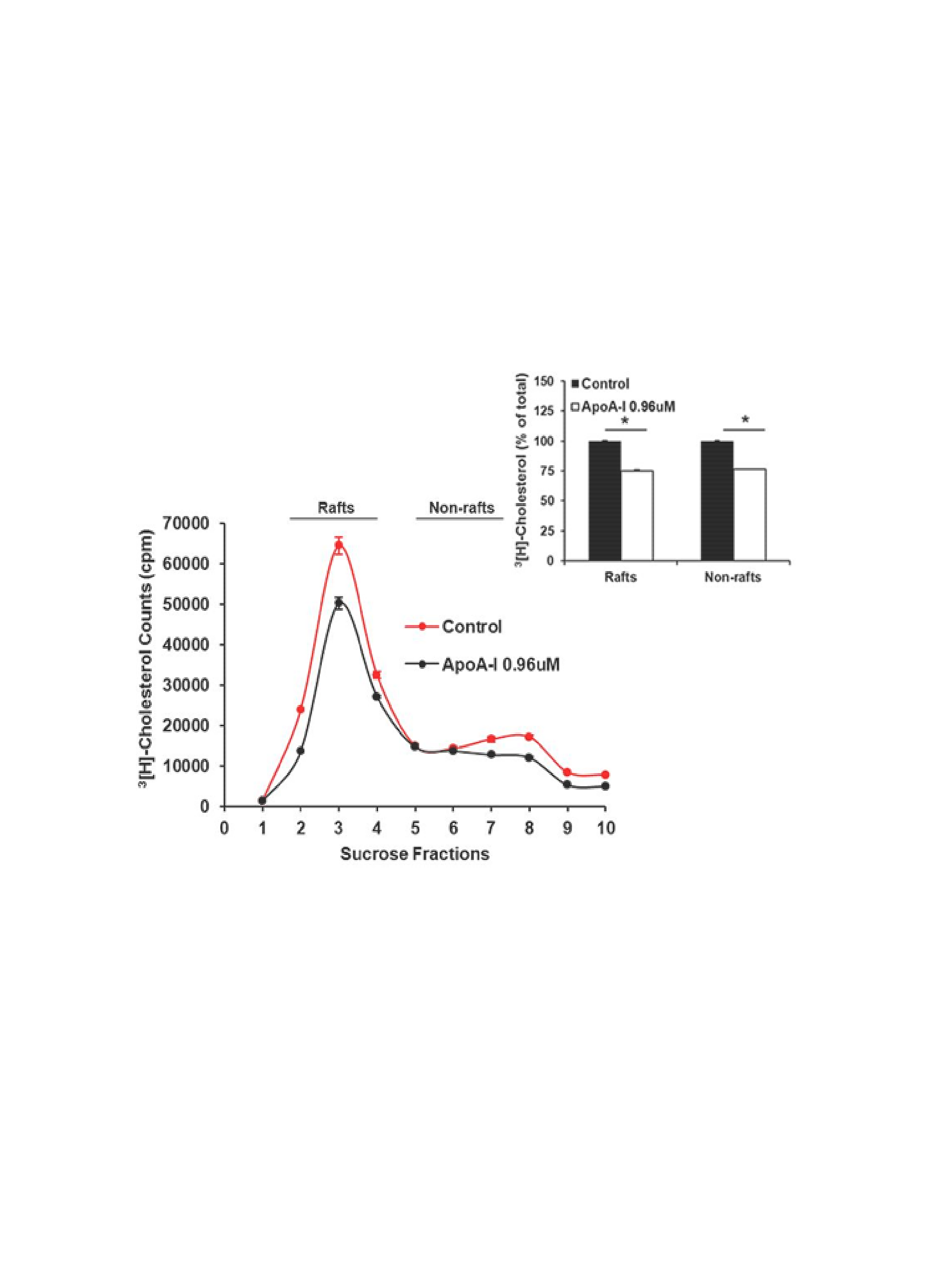

Supplement: S4 Fig — BHK-ABCA1 cells were labeled with 3[H]cholesterol or 3[H]choline for 48 h, followed by stimulation with mifepristone for 18–20h as described in experimental procedures in the online-only Supplementary appendix, S1 Appendix. Cells were then incubated for 45 min with apo A-I or CS-6253. (TIF) [file pone.0131997.s005.tif]

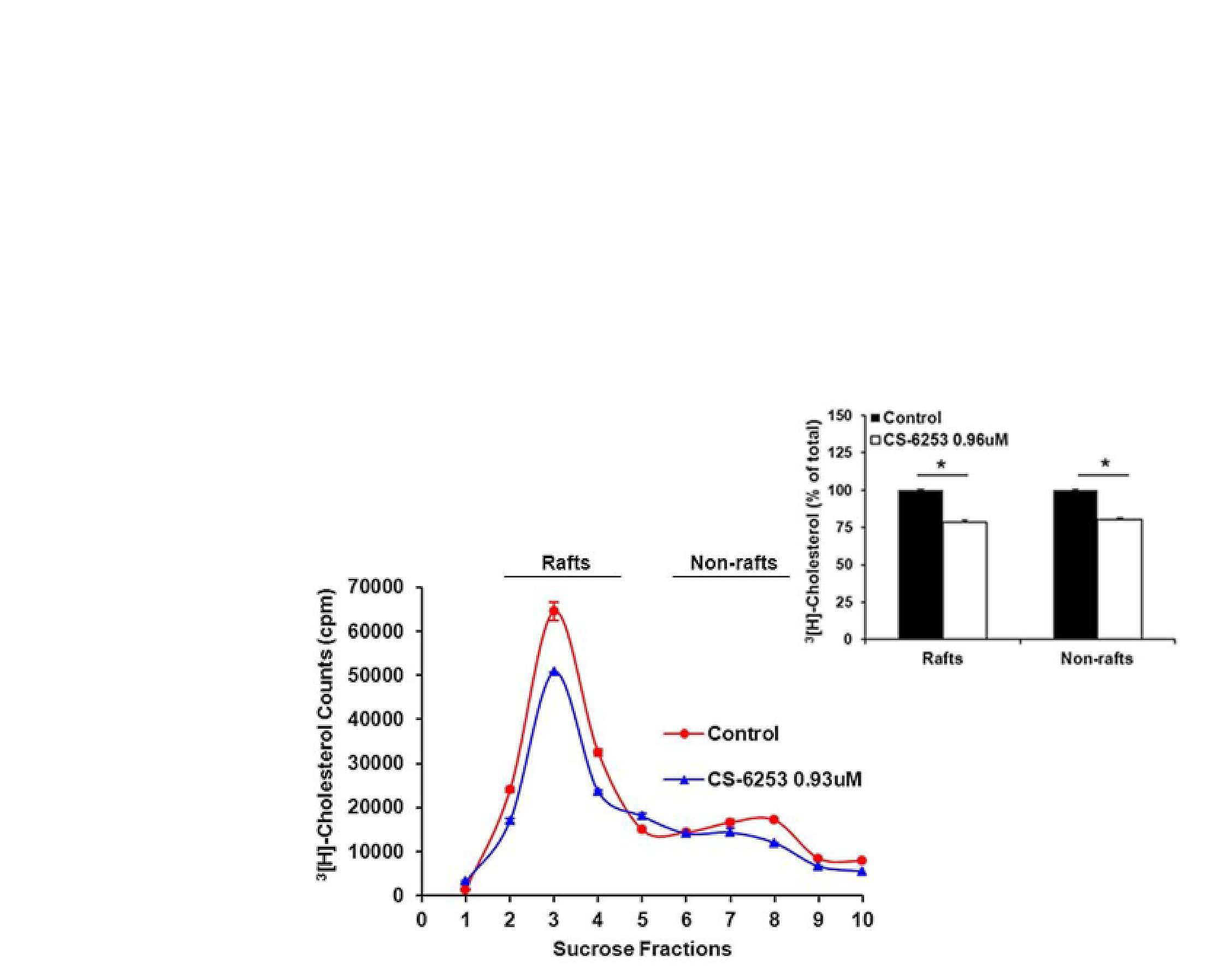

Supplement: S5 Fig — BHK-ABCA1 cells were labeled with 3[H]cholesterol or 3[H]choline for 48 h, followed by stimulation with mifepristone for 18–20h as described in experimental procedures in the online-only Supplementary appendix, S1 Appendix. Cells were then incubated for 45 min with apo A-I or CS-6253. (TIF) [file pone.0131997.s006.tif]

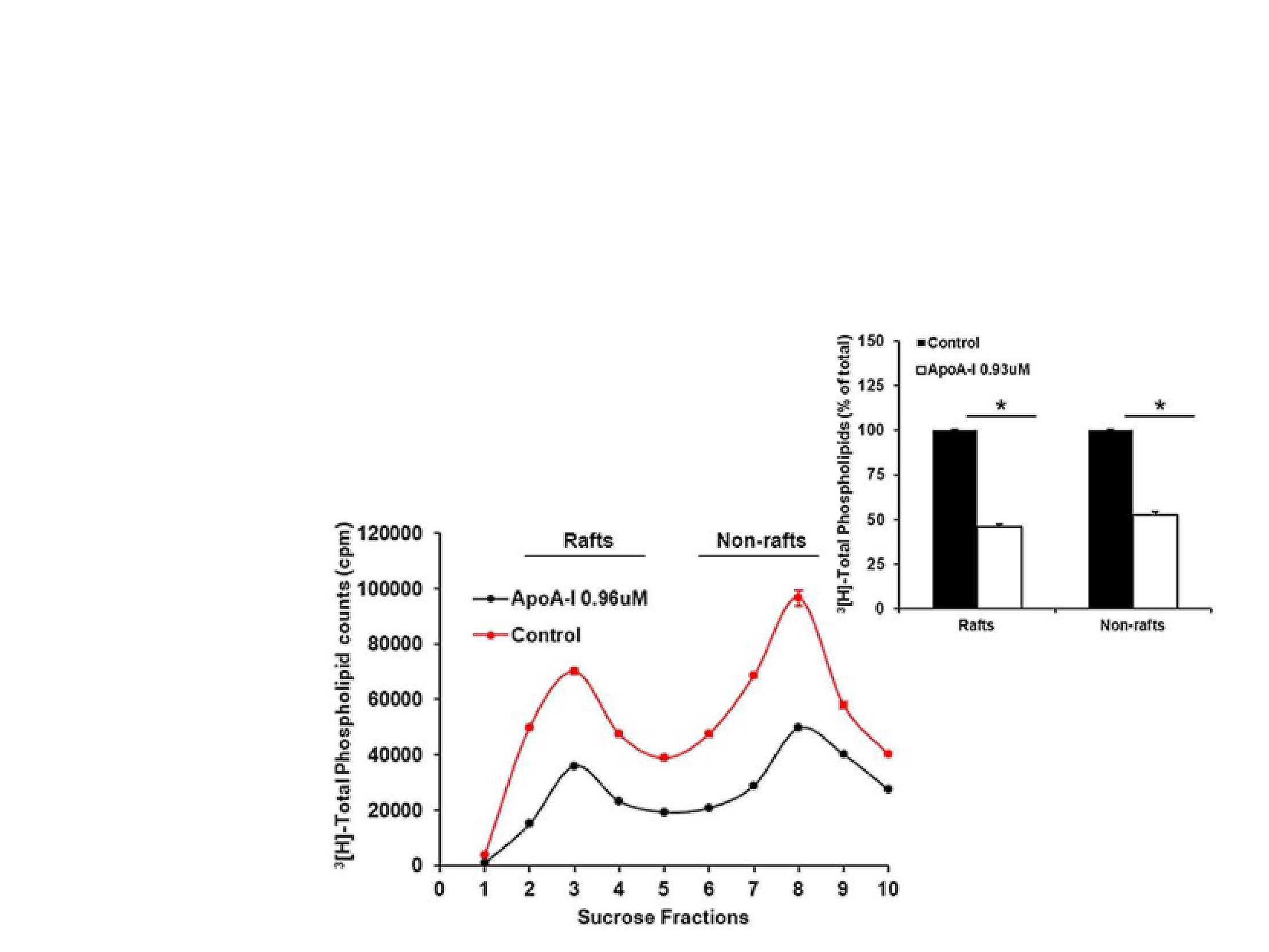

Supplement: S6 Fig — BHK-ABCA1 cells were labeled with 3[H]cholesterol or 3[H]choline for 48 h, followed by stimulation with mifepristone for 18–20h as described in experimental procedures in the online-only Supplementary appendix, S1 Appendix. Cells were then incubated for 45 min with apo A-I or CS-6253. (TIF) [file pone.0131997.s007.tif]

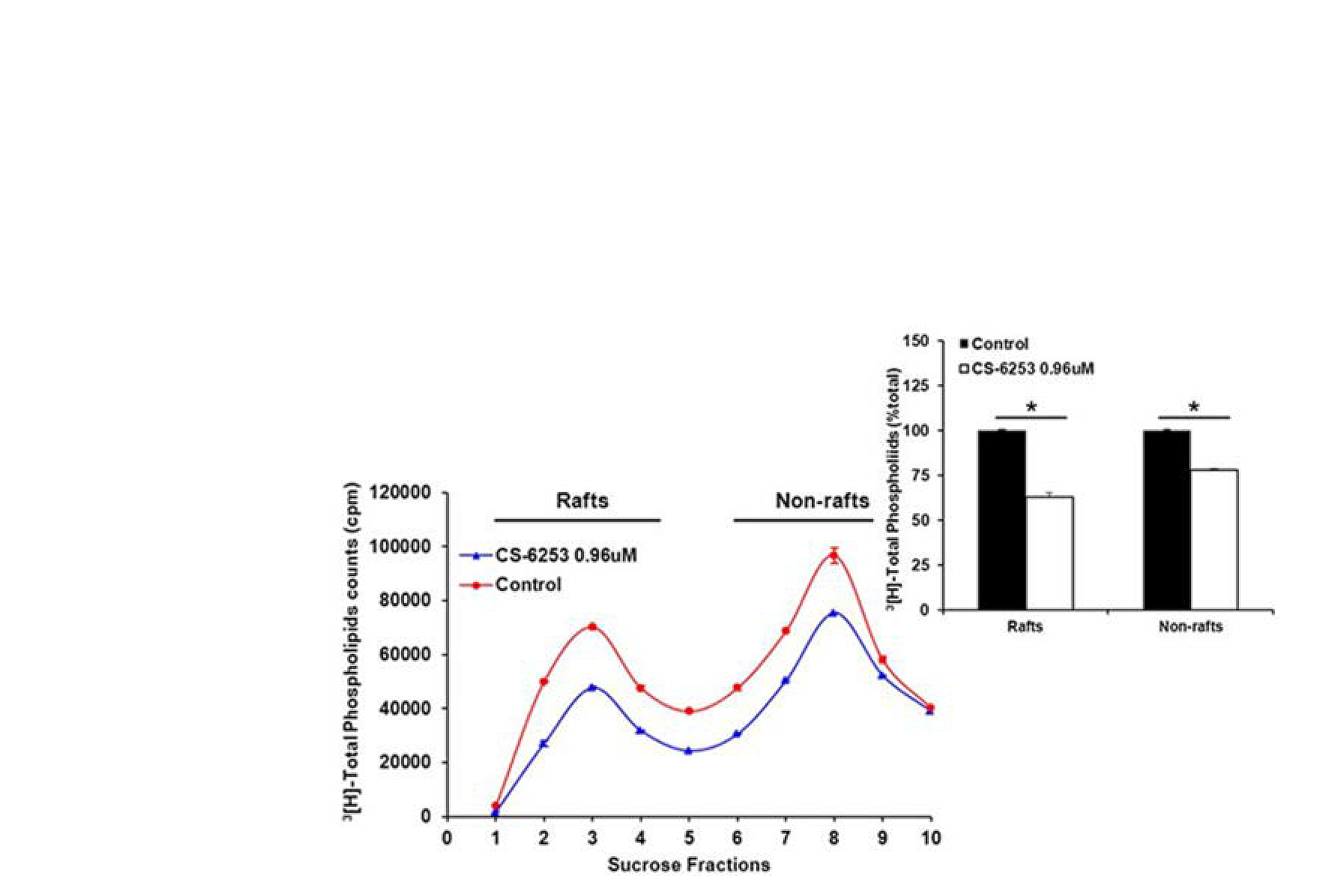

Supplement: S7 Fig — BHK-ABCA1 cells were labeled with 3[H]cholesterol or 3[H]choline for 48 h, followed by stimulation with mifepristone for 18–20h as described in experimental procedures in the online-only Supplementary appendix, S1 Appendix. Cells were then incubated for 45 min with apo A-I or CS-6253. After lipid extraction, 3[H]cholesterol in each fraction was assessed for radioactivity. Radioactivity appearing in fractions corresponding to raft (1–5) and nonraft (6–10) material was pooled, and desorption of 3[H]cholesterol and 3[H]choline, from raft versus nonraft in the presence of apo A-I or CS-6253 was expressed as a percentage of control (100%, in the absence of acceptor) (S4 to S7 inset). Results shown are representative of three independent experiments. *P < 0.05 by Student's t-test. (TIF) [file pone.0131997.s008.tif]

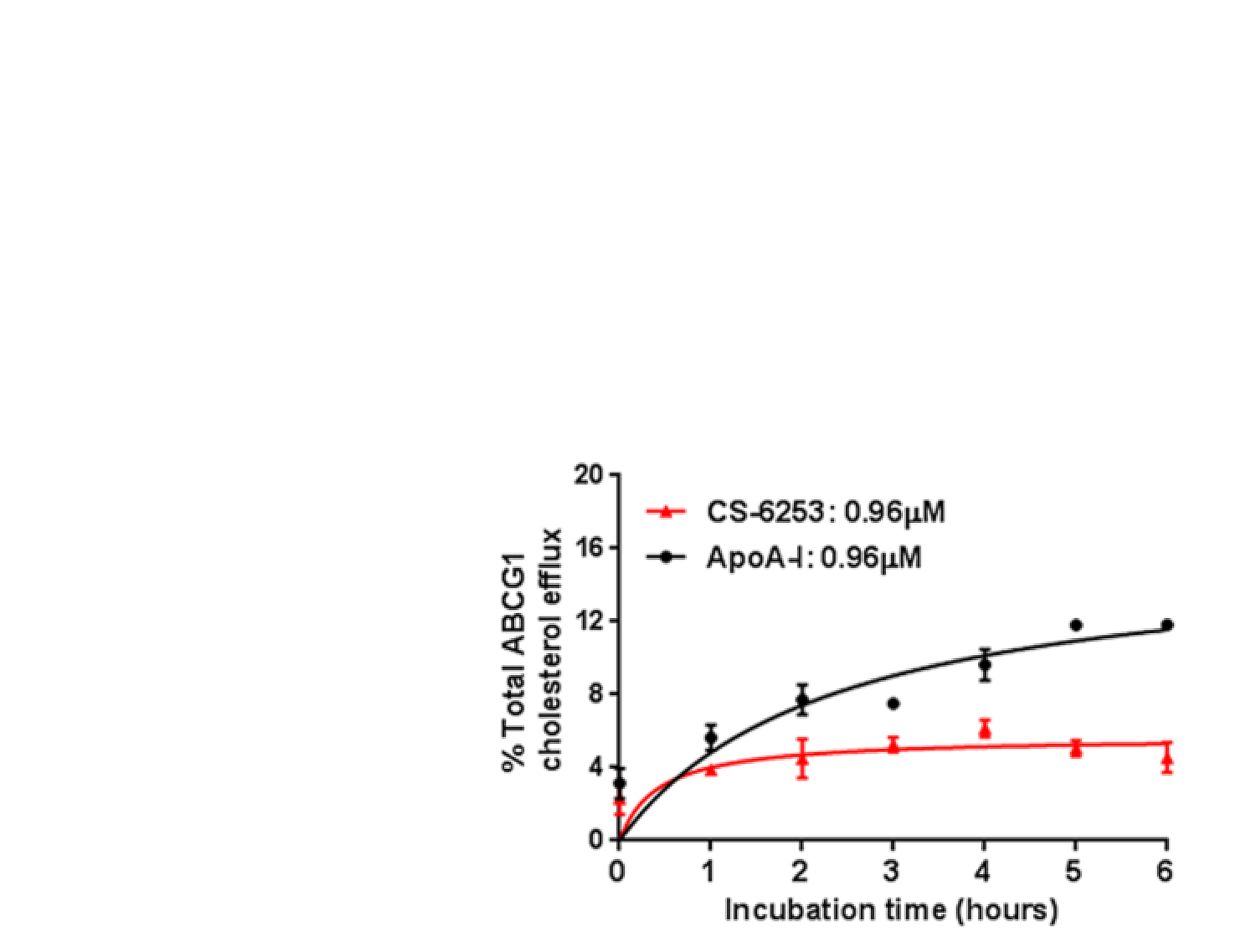

Supplement: S8 Fig — Unlabeled BHK cells expressing ABCA1 were treated with 0.96 μM lipid free apo A-I or CS-6253 for 18 h as described in experimental procedures in the online-only Supplementary appendix, S1 Appendix. Then the medium was collected and added to 3[H]cholesterol-labeled ABCG1 expressing cells in a time-dependent fashion (0–6h), and assayed for cholesterol efflux (only 6h time point is shown). (TIF) [file pone.0131997.s009.tif]

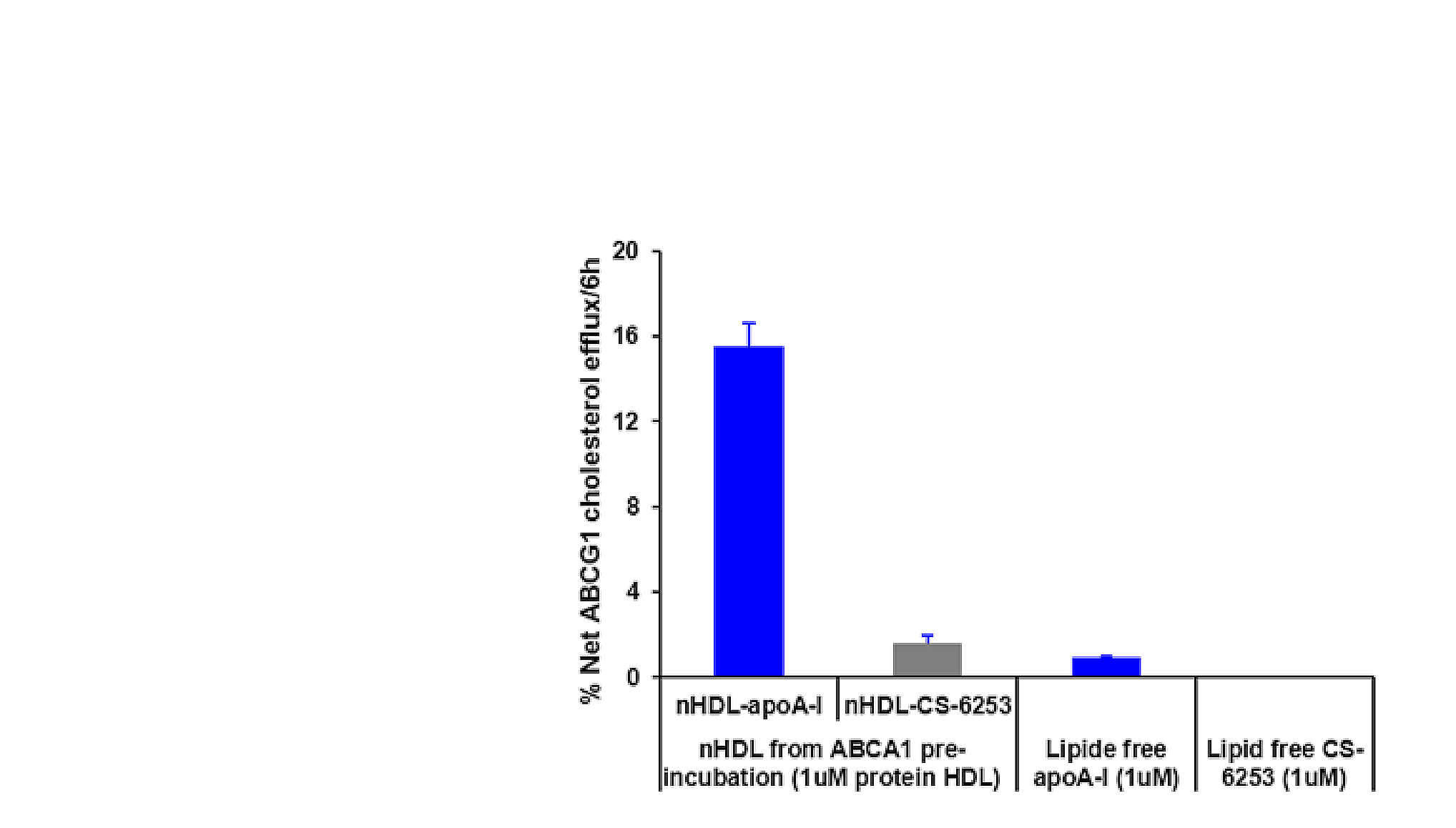

Supplement: S9 Fig — Unlabeled ABCA1 expressing cells were treated with increasing concentrations of apo A-I or CS-6253 for 18 h. The medium from unlabeled ABCA1 cells treated as described in experimental procedures, centrifuged to remove cellular debris, added to 3[H]cholesterol-BHK ABCG1 expressing cells for 6 h, and assayed for efflux as described in the online-only Supplementary appendix, S1 Appendix. Lipid free peptide and apo A-I are also tested against ABCG1 expressing cells for 6h cholesterol efflux. (We selected 1 μM nHDL mimetic or nHDL-apo A-I or lipid free peptide and apo A-I for the figure). Kinetic parameters for ABCG1-mediated cholesterol efflux to nHDL-apo A-I or CS-6253 are as follows: nHDL-Apo A-I: Km = 2.63±0.38 μg/ml (0.10±0.01 μM), Vmax = 16.23±0.61% efflux/6h, and relative catalytic efficiency: Vmax/Km = 6.17. nHDL-CS-6253: Km = 2.59±0.65 μg/ml (0.76±0.18 μM), Vmax = 4.61±0.65% efflux/4h, and relative catalytic efficiency: Vmax/Km = 1.77. Kinetic parameters for ABCG1-mediated cholesterol efflux to lipid free peptide and apo A-I are as follows: Apo A-I: Km = 1.7±0.50 μg/ml (0.06 ± 0.01 μM), Vmax = 0.96 ± 0.01% efflux/6h, and relative catalytic efficiency: Vmax/Km = 0.56. CS-6253: Km = 8.33±4.36 μg/ml (0.18±0.15 μM), Vmax = 0.99±0.19% efflux/6h, and relative catalytic efficiency: Vmax/Km = 0.11. Efflux of 3[H]cholesterol is shown as means ± SD of triplicate experiments. (TIF) [file pone.0131997.s010.tif]

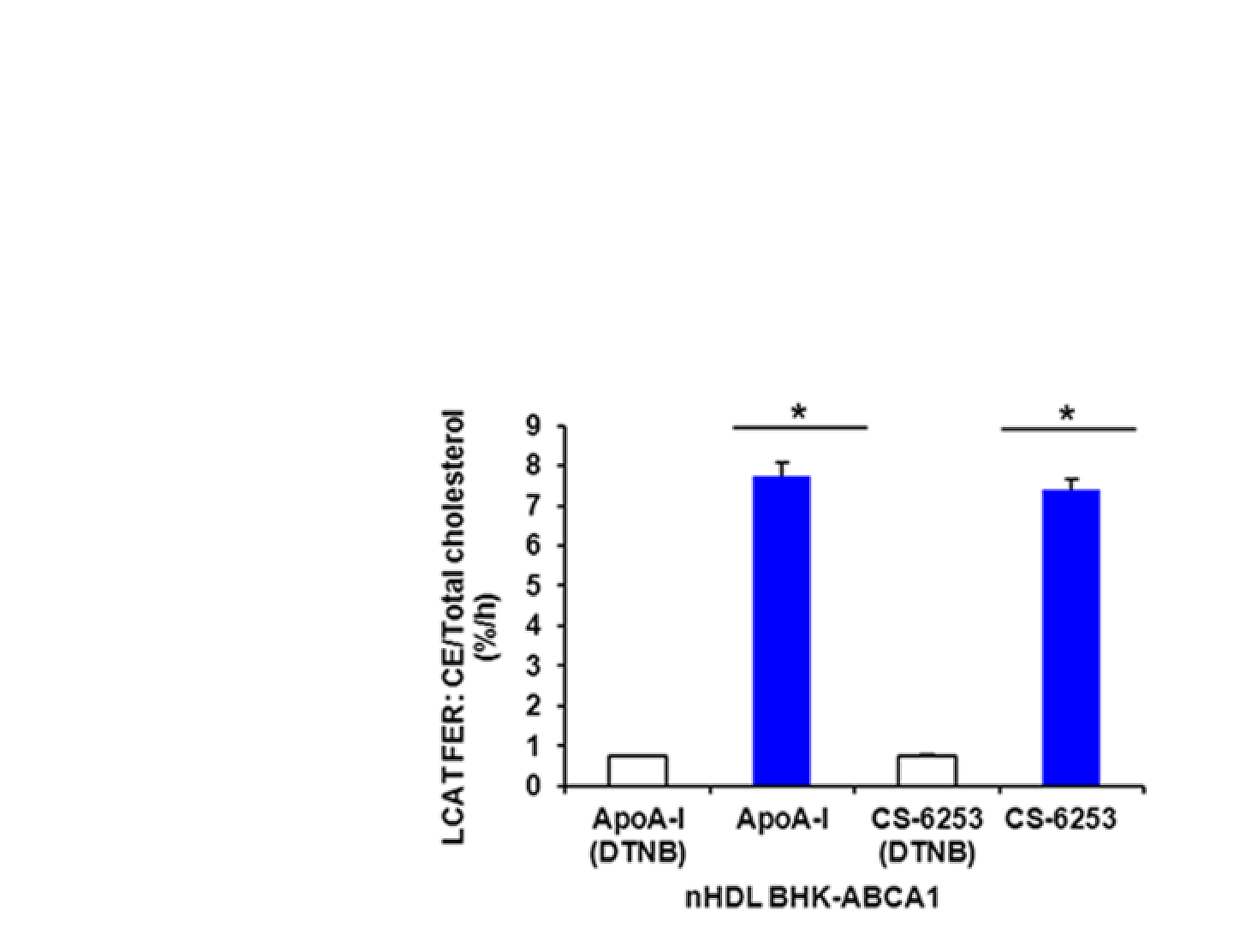

Supplement: S10 Fig — nHDL-CS-6253 particles were labelled with cell derived 3[H]cholesterol and incubated with a normolipidemic plasma at a ratio of (1μg peptide: 10μg plasma apo A-I). LCAT activity was determined after incubation for 1h at 37°C in the presence or absence of 2 mM DTNB. After lipid extraction, 3[H]cholesterol (unesterified) and 3[H]CE from plasma were separated by TLC and assayed for radioactivity as described in the online-only Supplementary appendix, S1 Appendix. LCAT activity was measured as CE divided by cholesterol/h. Results are mean (±SD) of triplicate experiments. *P<0.05. (TIF) [file pone.0131997.s011.tif]

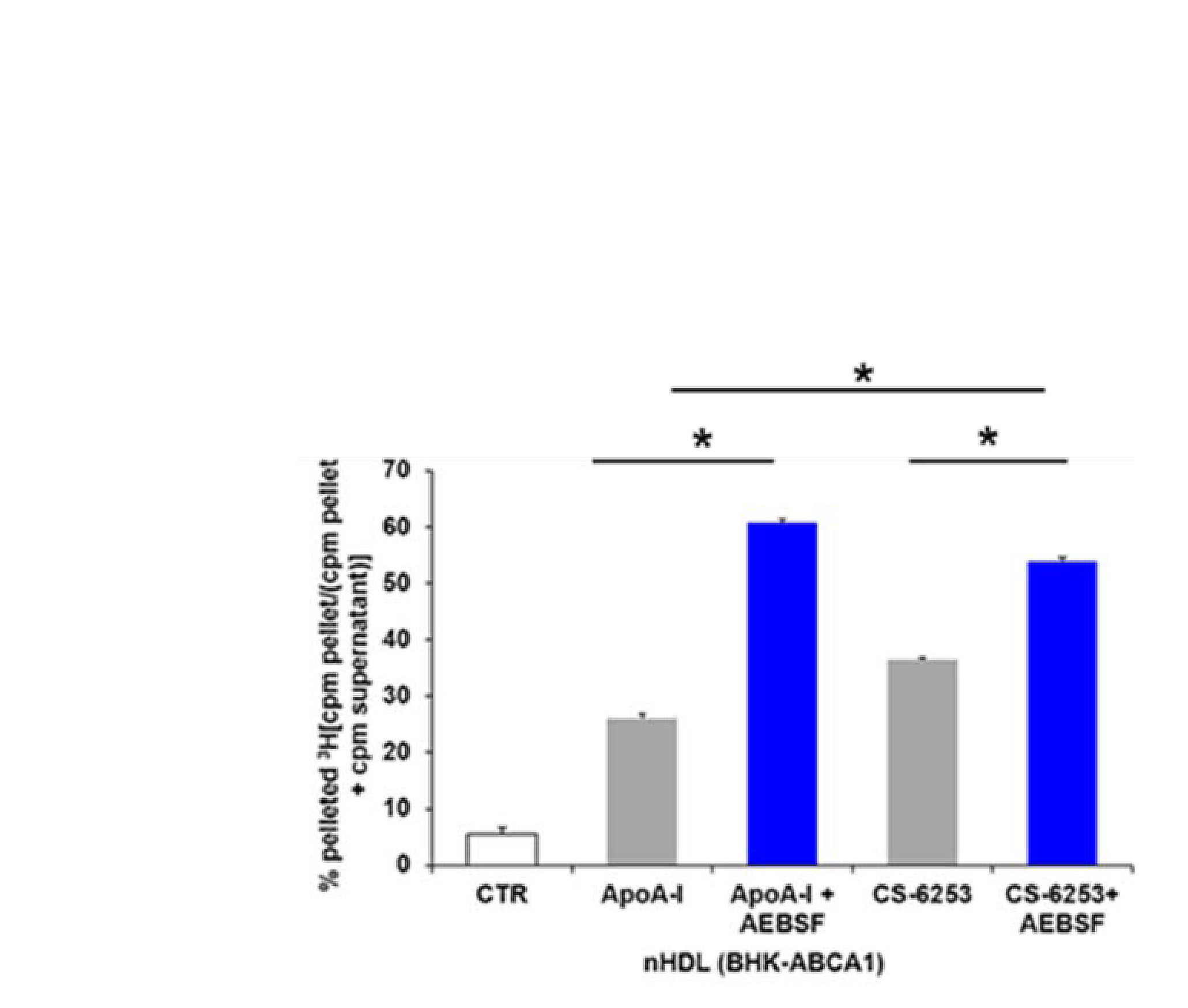

Supplement: S11 Fig — Radiolabelled 3[H]-choline nascent–like HDL lipoprotein apo A-I, CS-6253 or were incubated with normolipidemic plasma for 6 h at 37°C in the presence or the absence of AEBSF as indicated in methods in the online-only Supplementary appendix, S1 Appendix. After incubation, apoB was precipitated by PEG. ApoB containing particles fractions were dialyzed and PLTP transfer is calculated between nHDL and apoB fractions. Control (open bar) consists of radiolabelled particles alone in PBS+BSA. Results are mean (±SD) of triplicate experiments. *P<0.05. (TIF) [file pone.0131997.s012.tif]
